# *CFTR*-mediated monocyte/macrophage dysfunction revealed by cystic fibrosis proband-parent comparisons

**DOI:** 10.1172/jci.insight.152186

**Published:** 2022-03-22

**Authors:** Xi Zhang, Camille M. Moore, Laura D. Harmacek, Joanne Domenico, Vittobai Rashika Rangaraj, Justin E. Ideozu, Jennifer R. Knapp, Katherine J. Woods, Stephanie Jump, Shuang Jia, Jeremy W. Prokop, Russell Bowler, Martin J. Hessner, Erwin W. Gelfand, Hara Levy

**Affiliations:** 1 Data Science program, Weinberg College of Arts and Sciences, Northwestern University, Evanston, Illinois, USA.; 2 Division of Pediatric Pulmonary Medicine, Department of Pediatrics, and; 3 Center for Genes, Environment and Health, National Jewish Health, Denver, Colorado, USA.; 4 Division of Pulmonary & Sleep Medicine, Ann & Robert H. Lurie Children’s Hospital of Chicago, Chicago, Illinois, USA.; 5 Genomic Medicine, Genomics Research Center, AbbVie, North Chicago, Illinois, USA.; 6 Department of Pediatrics, Medical College of Wisconsin, Milwaukee, Wisconsin, USA.; 7 Department of Pediatrics, National Jewish Health, Denver, Colorado, USA.; 8 Max McGee Center for Juvenile Diabetes, Children’s Research Institute, Children’s Hospital of Wisconsin, Milwaukee, Wisconsin, USA.; 9 Department of Pediatrics and Human Development, College of Human Medicine, Michigan State University, Grand Rapids, Michigan, USA.; 10 Division of Pulmonary and Critical Care Medicine, Department of Medicine, and; 11 Division of Cell Biology, Department of Pediatrics, National Jewish Health, Denver, Colorado, USA.; 12 Division of Immunology, Microbiology and Pediatrics, Department of Pediatrics, University of Colorado School of Medicine, Denver, Colorado, USA.

**Keywords:** Pulmonology, Epigenetics, Macrophages, Monocytes

## Abstract

Cystic fibrosis (CF) is an inherited disorder caused by biallelic mutations of the CF transmembrane conductance regulator (*CFTR*) gene. Converging evidence suggests that CF carriers with only 1 defective *CFTR* copy are at increased risk for CF-related conditions and pulmonary infections, but the molecular mechanisms underpinning this effect remain unknown. We performed transcriptomic profiling of peripheral blood mononuclear cells (PBMCs) of CF child-parent trios (proband, father, and mother) and healthy control (HC) PBMCs or THP-1 cells incubated with the plasma of these participants. Transcriptomic analyses revealed suppression of cytokine-enriched immune-related genes (*IL-1β*, *CXCL8*, *CREM*), implicating lipopolysaccharide tolerance in innate immune cells (monocytes) of CF probands and their parents. These data suggest that a homozygous as well as a heterozygous *CFTR* mutation can modulate the immune/inflammatory system. This conclusion is further supported by the finding of lower numbers of circulating monocytes in CF probands and their parents, compared with HCs, and the abundance of mononuclear phagocyte subsets, which correlated with *Pseudomonas aeruginosa* infection, lung disease severity, and CF progression in the probands. This study provides insight into demonstrated *CFTR*-related innate immune dysfunction in individuals with CF and carriers of a *CFTR* mutation that may serve as a target for personalized therapy.

## Introduction

Cystic fibrosis (CF) is a hereditary disorder caused by mutations in the CF transmembrane conductance regulator (*CFTR*) gene. However, both intrinsic and extrinsic variables directly and/or indirectly associated with *CFTR* likely influence the course of CF, particularly the immune/inflammatory phenotype of CF lung disease (1). Many pathological hallmarks of CF such as chronic airway infection, persistent inflammation (2), and defective mucociliary clearance are consequences of deficient or defective CFTR protein in airway epithelial cells. Over time, these cells fail to eradicate pulmonary pathogens, which in turn contribute to a mucosal immunodeficiency syndrome (3–5). In previous studies, plasma from individuals with CF compromised biological signaling and dysregulated mRNA and miRNA interactions in peripheral blood mononuclear cells (PBMCs), suggesting an impaired response in the circulating immune cells of patients with CF (6–10).

In support of this notion, innate immune cells — represented primarily by monocytes, macrophages, and dendritic cells (DCs), which are initially recruited to combat bacterial pathogens — are dysregulated in CF airways (10). Although the significance and molecular basis of this dysregulation remain unclear, mutations in *CFTR* can not only affect the innate immune function of airway epithelial cells but also alter the innate immunity that contributes to recurrent and progressive infection in CF (11–13). Previous work showed that CF F508del/F508del (homozygous) murine macrophages have a defective response and reduced cytotoxic activity against the bacterial pathogen *Pseudomonas aeruginosa* (*P. aeruginosa*), which is prevalent in patients with CF (14, 15). While infecting bacteria exploit these defects in macrophage function (16, 17), treatment with the CFTR modulator ivacaftor improves macrophage-mediated cytotoxicity (18). Further, long noncoding (lncRNAs) have key roles in regulating the innate immune response to *P. aeruginosa* in CF, combining with other regulatory mechanisms to alter the expression of immune/inflammatory genes within monocytes and macrophages (19–22). However, the genes and regulatory pathways involved in this immune dysregulation in individuals with CF have not been well characterized.

Because CF is an autosomal recessive disorder with 2 defective *CFTR* copies, heterozygous carriers are typically considered healthy. However, CF carriers have an increased risk for a broad range of conditions affecting multiple organ systems, including asthma and airway infections (23, 24). Several studies reported familial clusters of pulmonary infections with nontuberculous mycobacteria, suggesting genetic risk factors, including *CFTR* mutations (25, 26). The observations that both CF carriers (CF parents) and patients with CF are at higher risk of CF-related conditions than people without *CFTR* mutations suggest that distinct *CFTR*-related mechanisms are at play in both heterozygous and homozygous individuals (27, 28).

**Figure 1:**
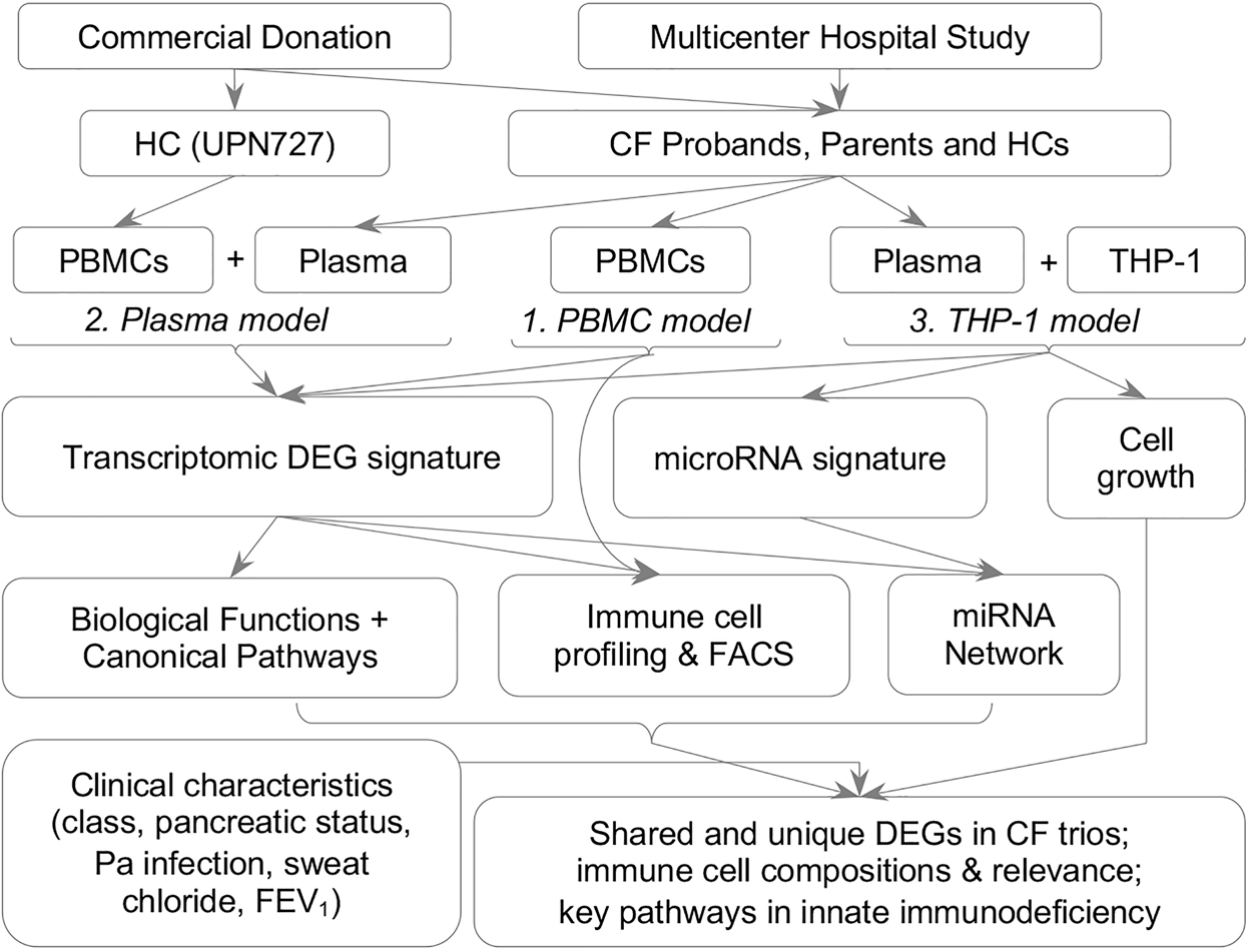
Schematic of main study procedures. DEGs, differentially expressed genes; FEV_1_, forced expiratory volume in 1 second.

To understand the distinct molecular features and pathways contributing to known immune phenotypes in individuals with CF and carriers, we assembled a cohort of parent-child trios (CF proband, father, and mother) and 20 unrelated healthy controls (HCs) without *CFTR* mutations (Figure 1 and Table 1). We identified *CFTR*-related immune suppression in each trio subgroup through transcriptomic profiling using 3 cellular models: (i) a PBMC model; (ii) a plasma model, where the donors’ PBMCs act as reporters of the immune microenvironment and compromised immune/inflammatory conditions in individuals with CF; and (iii) a THP-1 cell model, where THP-1 cells are incubated with plasma, replicating the effects of the immune microenvironment on monocyte and macrophage function. Utilizing a previously established cell composition deconvolution method (8), we observed gene suppression in innate immune cells from *CFTR*-mutated PBMCs and from healthy PBMCs incubated with CF plasma. We determined that the abundance of mononuclear phagocytes correlated with CF clinical characteristics. Plasma samples from individuals within the trios were used in ex vivo cultures of THP-1 monocytes and macrophages to further characterize the transcriptomic profiles unique to patients with CF and the profiles CF trios shared. Consistent with these findings, our gene set enrichment analyses suggested likely impaired responsiveness of CF PBMCs and monocytes to lipopolysaccharide (LPS), an integral component not only of the *P. aeruginosa* cell envelope, which is the predominant organism in oropharyngeal cultures within the CF airway, but also of other Gram-negative opportunistic pathogens. This approach of blood-based profiling and association with CF disease state provides important clues in understanding the vulnerability of carriers of *CFTR* mutations.

## Results

### Profiling of PBMCs reveals significant downregulation of immune/inflammatory markers.

**Figure 2:**
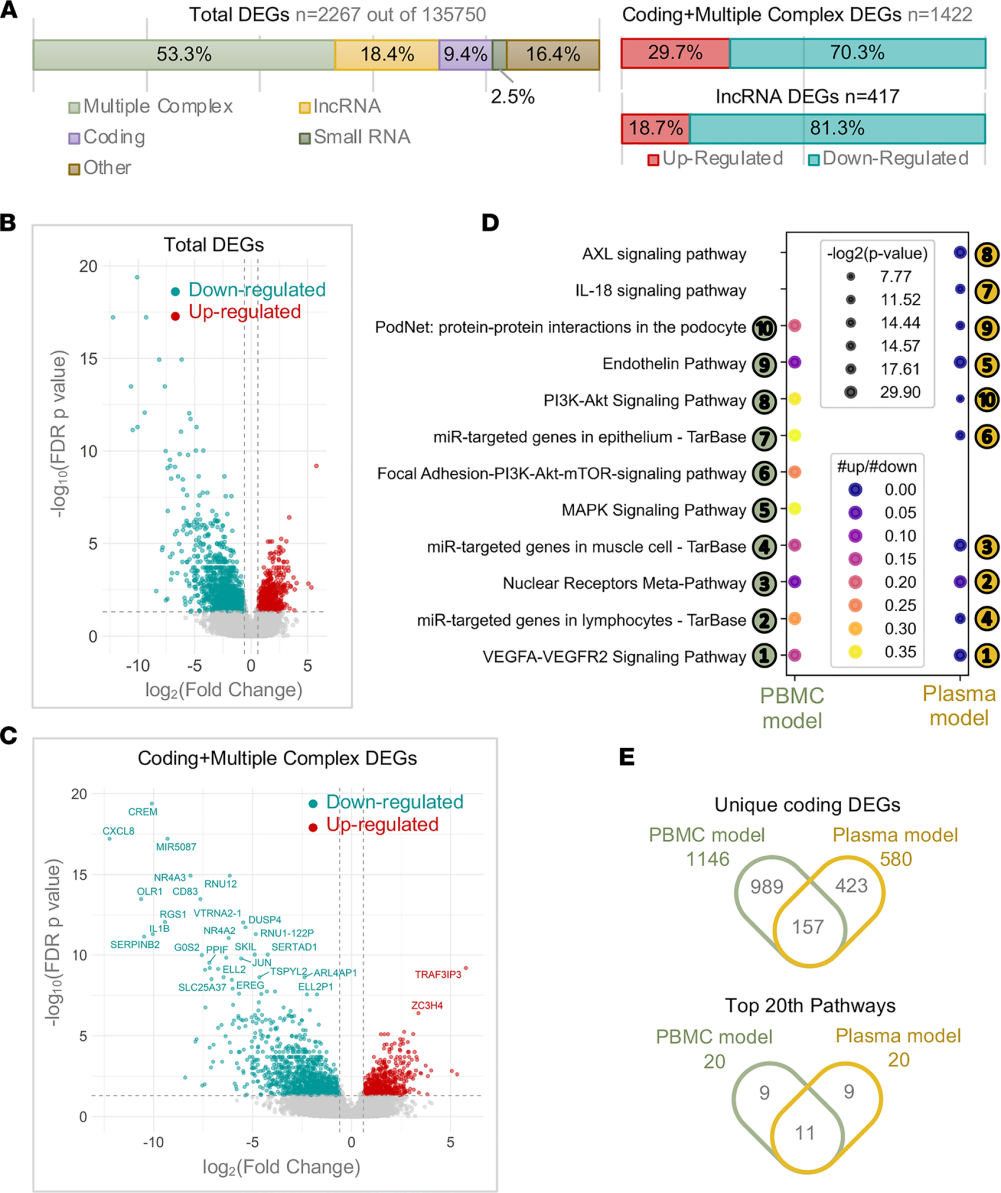
Immune-associated genes and pathways are significantly downregulated in CF PBMCs compared with HCs. (**A**) Transcripts differentially expressed between CF probands (*n* = 14) and HCs (*n* = 8) in the PBMC model, divided into categories according to locus type. We identified differentially expressed transcripts that displayed a more than 2-fold change in expression level and an FDR-adjusted *P* < 0.05. (**B** and **C**) Volcano plots of differentially expressed transcripts. (**D**) Bubble plot of the top 10 significant pathways in WikiPathways, ranked by the number of genes in the pathway. For the plasma model: CF probands (*n* = 24), HCs (*n* = 20). (**E**) Venn diagrams showing the numbers and overlap of unique genes (upper) and top 20 pathways (lower) for CF probands versus HCs in the PBMC and plasma models.

To identify blood-based gene predictors that are clinically useful, we collected PBMCs from CF probands (*n* = 14), either 1 of the parents, mothers (*n* = 14), and HCs (*n* = 8). We then measured transcriptional expression in these cells with whole-transcriptome arrays (Figure 1 and Table 1, PBMC model; Methods). Differential expression analyses revealed that a notable number of transcripts (2267 out of 135,750) were differentially expressed between CF probands and HCs (Figure 2A, left). More than half of these genes/transcripts (DEGs) encode proteins (*n* = 1422, including 213 from the “coding” category and 1209 from the “multiple complex” category, where “multiple complex” is defined as a transcript reported in multiple locus types; see Figure 2A, left; Supplemental Table 1; supplemental material available online with this article; https://doi.org/10.1172/jci.insight.152186DS1). Among these DEGs, more than 70% of coding RNAs (including multiple complex) and more than 80% of lncRNAs were downregulated in CF proband PBMCs (Figure 2A, right).

We identified DEGs that showed large fold changes (log_2_ fold changes, –15 to 5) in expression in proband PBMCs relative to HC PBMCs (Figure 2B). Among these were genes that encoded key cytokines (e.g., *IL-1β*), chemokines (e.g., *CXCL8*), nuclear receptors (e.g., nuclear receptor subfamily 4 group A member 3, *NR4A3*), and modulators of immune signaling pathways (e.g., cAMP responsive element modulator, *CREM*) (Figure 2C). We identified fewer upregulated protein-coding genes than downregulated genes in the proband PBMCs, including genes that code for proteins that interact with TNF receptor-associated factor (*TRAF3IP3*) and posttranscriptional regulators (zinc finger domain-containing protein, *ZC3H4*; see Figure 2C). Most of the top-ranking pathways enriched in the DEG list were cytokine-related signaling pathways (29); each was downregulated in CF proband PBMCs compared with HC PBMCs (ratio of upregulated and downregulated gene numbers < 1; see Figure 2D). Thus, the observed imbalance between up- and downregulated transcript profiles in PBMCs of CF probands versus PBMCs of HCs provides further evidence that the immune/inflammatory pathways are aberrant in CF.

### CF probands and parents share similar transcriptomic features in PBMCs.

To determine the transcriptomic signatures that were unique to and shared among the subgroups within our CF proband-parent trios, we performed transcriptomic profiling using both the PBMC and plasma models consisting of healthy donor PBMCs cultured with plasma from the CF probands or either parent (Figure 1 and Table 1). The CF proband-associated transcriptomic profiles from the PBMC and plasma models shared common DEGs (Figure 2E, Supplemental Figure 1, and Supplemental Table 2) and functional pathways (Figure 2E and Supplemental Table 3), again suggesting that compared with HCs, the CF probands consistently exhibit extensive alterations leading to abnormal cytokine and chemokine profiles.

**Figure 3:**
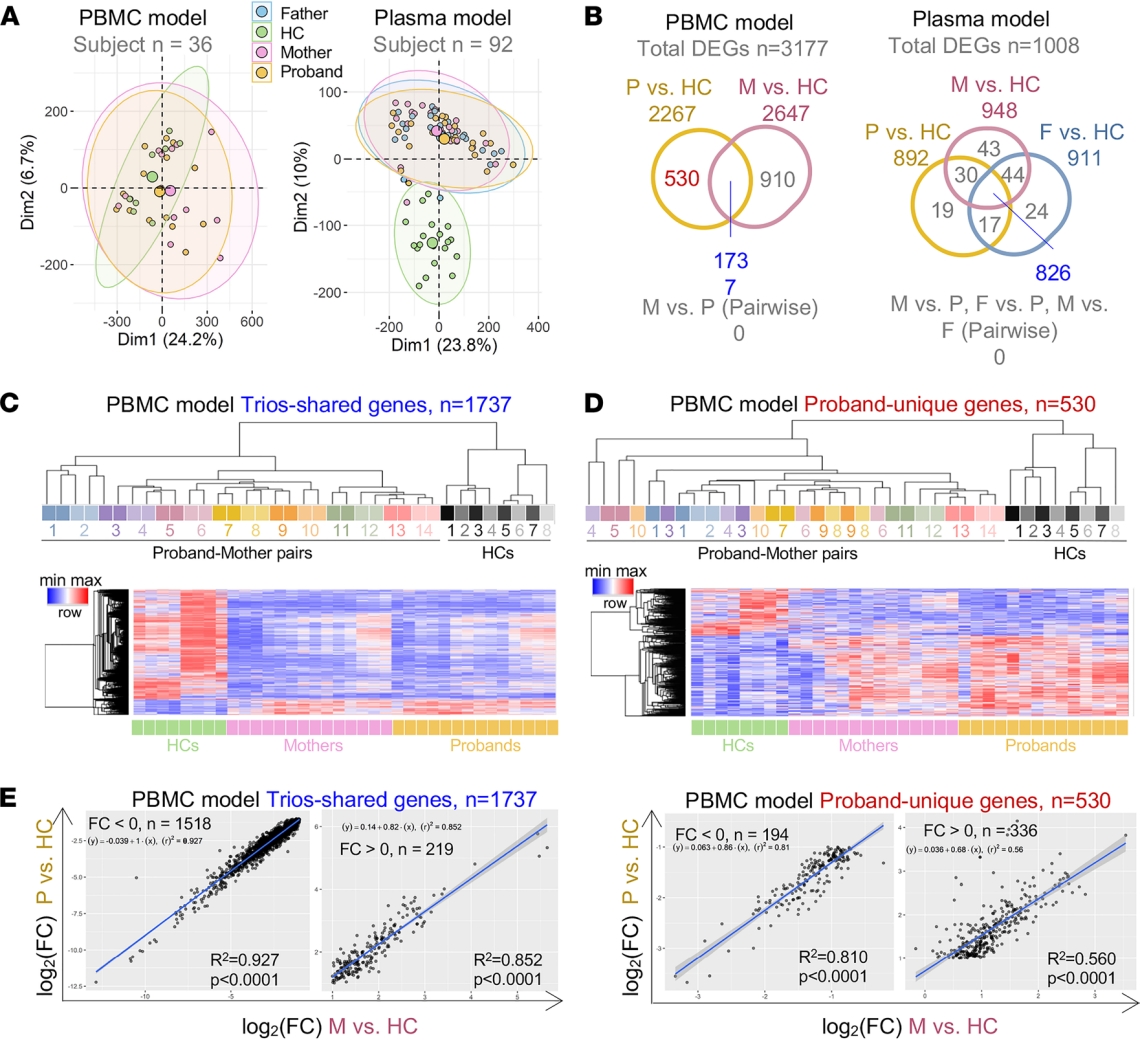
CF carriers and probands share highly similar transcriptomic profiles in PBMCs. (**A**) Principal component analysis (PCA) of data from the PBMC (left; *n* = 36) and plasma models (right; probands, *n* = 92). (**B**) Venn diagrams of the numbers and overlap of DEGs from the PBMC and plasma models; comparisons are as indicated. Trio-shared and proband-unique genes are highlighted in blue and red, respectively. (**C** and **D**) Top, hierarchical clustering of study participants; bottom, heatmap of the expression of trio-shared and proband-unique genes in the PBMC model. (**E**) Correlation scatterplots of the fold change (log_2_) of the 2 indicated comparisons of the expression of trio-shared (left) and proband-unique genes (right) from the PBMC model. The *P* value and *R*^2^ (square of the correlation coefficient) were produced by a Pearson’s correlation analysis. The linear regression line and its equation were generated from a simple linear regression analysis. P, proband; F, father; M, mother; FC, fold change.

Principal component analysis (PCA) of transcriptomic profiles from the plasma model (*n* = 92 in 4 subgroups; Table 1) revealed overlap among the CF proband and parent subgroups within the CF trios, which clustered separately from the HCs (Figure 3A and Supplemental Figure 2A, left). In contrast, PCA of transcriptomic profiles from the PBMC model (*n* = 36 in 3 groups) indicated no separation between the CF trios and HC subgroup clusters, although the HC cluster was not as broadly distributed as the proband and mother clusters (Figure 3A and Supplemental Figure 2A, right). There was substantial overlap in the DEGs from CF probands versus HCs and from CF parents versus HCs in both the PBMC model (*n* = 1737) and the plasma model (*n* = 826; see Figure 3B). Interestingly, we did not detect any significant DEGs when comparing CF probands with parents in either model. In the PBMC model, we identified DEGs shared among CF probands and CF mothers (trio-shared genes; *n* = 1737) as well as DEGs that were unique to CF probands (proband-unique genes; *n* = 530); however, we did not identify DEGs unique to CF mothers (Figure 3B and Supplemental Tables 4 and 5).

We next performed hierarchical clustering predicting homogeneous groups among trio participants. Notably, only the dendrogram generated using mother/proband-shared genes from the PBMC model (*n* = 1737) organized participants into groups in accordance with their family structures (Figure 3C, upper). We did not consistently observe this familial grouping in the clustering developed using proband-unique genes in the PBMC model (Figure 3D, upper) or trio-shared genes in the plasma model (Supplemental Figure 2B). Overall, these results suggest that the mother/proband-shared genes in the PBMC model capture the relative homogeneity of gene expression across CF trio participants.

The resulting heatmaps revealed near-identical expression patterns of trio-shared genes across the CF trios (mothers and probands versus HCs; Figure 3C, lower panel). In contrast, we observed less similarity between mothers and probands in the proband-unique genes (Figure 3D, lower). We determined whether the trio-shared genes displayed a consistent direction and magnitude of expression in the trios relative to HCs. The correlation between the expression levels of shared genes in CF probands and their mothers was significant (*P* < 0.0001) and showed a strong linear relationship (upregulated: *R*^2^ = 0.927, downregulated: *R*^2^ = 0.852) (Figure 3E, left). The correlation of the expression of proband-unique genes remained significant (*P* < 0.0001) but showed a weaker linear relationship (upregulated: *R*^2^ = 0.81, downregulated: *R*^2^ = 0.56; see Figure 3E, right). Together, these transcriptomic profiling results indicate that parents and CF probands share similar expression patterns in the PBMC and plasma models.

### CF probands and parents share unique immune cell compositions.

**Figure 4:**
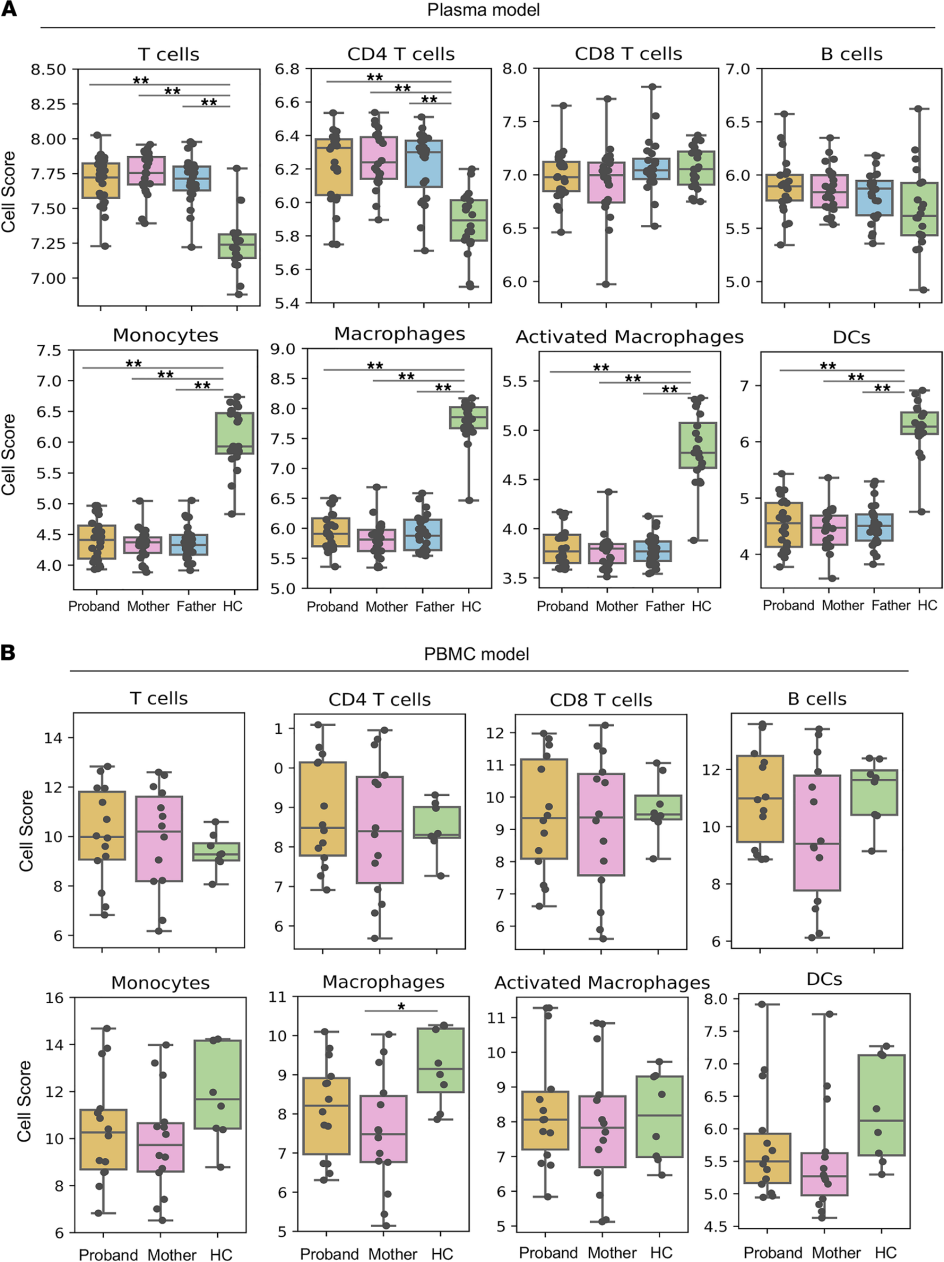
Immune cell composition differs between CF parent-child trios and HCs. Dot and box plots of the cell composition scores of 8 cell subsets in the (**A**) plasma model (probands, *n* = 24; parents/carriers, *n* = 48; HCs, *n* = 20) and (**B**) PBMC model (probands, *n* = 14; parents/carriers, *n* = 14; HCs, *n* = 8). Estimates of cell numbers in each cell subset were calculated for CF trios and HCs. The means were compared by paired and unpaired independent *t* test, as appropriate; and *P* values were adjusted using Holm-Šidák method to control the family-wise error rate; *P < 0.05, **P < 0.01.

We previously developed a cell composition deconvolution method to estimate immune cell type composition from gene expression data (8). Using this method, we found that myeloid cell subsets were less abundant while lymphoid cell subpopulations were more abundant in PBMCs incubated with CF proband plasma versus HC plasma (8). Here, we employed a similar deconvolution method (Methods and Supplemental Figures 3 and 4) to infer the immune cell compositions of CF probands and their parents in our plasma model with donor PBMCs. We estimated a significantly higher abundance of 2 out of 5 lymphoid cell subsets (total T cells and CD4^+^ T cells) and a significantly lower abundance of all 5 myeloid cell subsets (monocytes, macrophages, activated monocytes, activated macrophages, and DCs) in cells incubated with CF proband plasma than in cells incubated with HC plasma (Figure 4A and Supplemental Figure 5A). These differences were also observed when comparing results obtained with the plasma of CF parents and with HC plasma.

The immune cells in the plasma model were harvested from a single donor (identical genomic background), whereas the immune cells in the PBMC model were harvested from the patients with CF and HCs (yielding greater genomic heterogeneity; Table 1). We hypothesized that mutations in *CFTR* might lead to a phenotype that disrupts immune cell composition in CF PBMCs. Indeed, higher and lower cell numbers, respectively, were estimated in the lymphoid and myeloid cell subsets of PBMCs from CF probands versus HCs, but these differences were not statistically significant (Figure 4B and Supplemental Figure 5B). Macrophages were significantly less abundant in CF parents (*P* < 0.05) than in HCs (Figure 4B and Supplemental Figure 5C), but we did not observe a significant difference between CF probands and HCs. We confirmed these findings via flow cytometry: both total circulating monocytes (CD14^+^) and classical monocytes (CD14^+^CD16^−^) were less abundant in CF probands than in HCs (Supplemental Figure 5D). These results suggest that immune cell composition differs between CF trios and HCs, potentially identifying involvement of the monocyte/macrophage lineage in CF-related immunodeficiency.

### Phagocytic cell abundance is associated with CF disease severity and progression.

**Figure 5:**
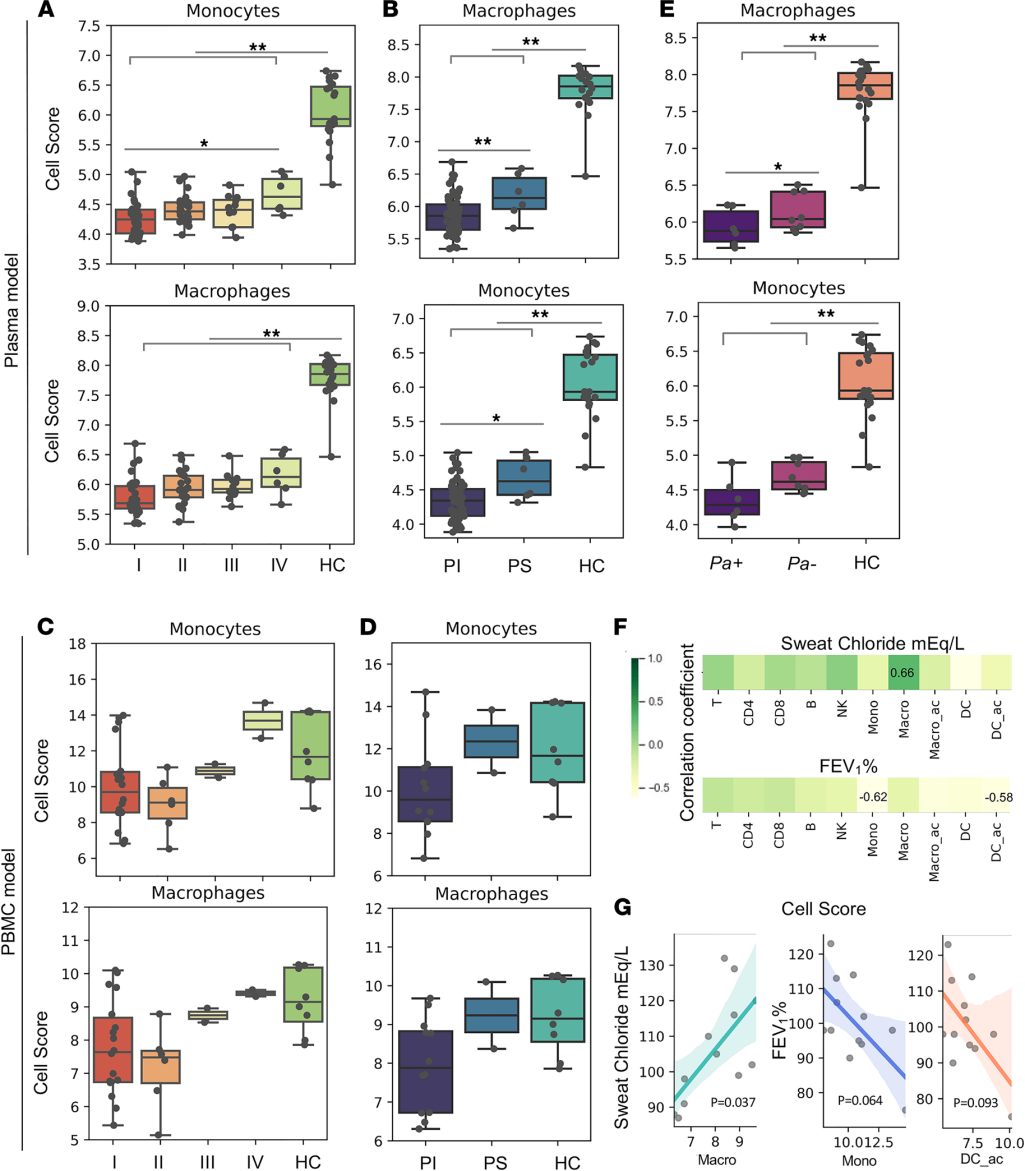
Compositions of monocytes and macrophages in the plasma and PBMC models are correlated with CF disease severity and progression. (**A**–**E**) Dot and box plots of cell composition scores (see Methods) of monocytes and macrophages in patients with CF grouped based on their (**A** and **C**) *CFTR* class, (**B** and **D**) pancreatic function, and (**E**) *P*. *aeruginosa* infection status. Estimations of cell numbers in each cell subset were compared between subgroups of patients with CF (**F** and **G**). Correlation analysis of cell abundance with sweat chloride and percent predicted FEV_1_. Plasma model (**A**, **B**, and **E**); PBMC model (**C**, **D**, **F**, and **G**). The means were compared by paired and unpaired independent *t* test, as appropriate; and *P* values were adjusted using Holm-Šidák method to control the family-wise error rate. The *P* value and *R* (correlation coefficient) were produced by a Pearson’s correlation analysis (normal distribution assumed). *P < 0.05, **P < 0.01. Mono, monocytes; Macro, macrophages; Macro_ac, activated macrophages; DC_ac, activated dendritic cells; PI, pancreatic insufficient; PS, pancreatic sufficient.

Mutations in *CFTR* are divided into 6 classes (I–VI) according to aspects of CFTR biogenesis, metabolism, and function (30). The clinical severity and progression of CF can be predicted by categorical attributes such as *CFTR* class, pancreatic sufficiency status, *P. aeruginosa* infection status, and numerical measures such as sweat chloride level and FEV_1_ (30–33). To associate our findings on immune cell composition in CF with clinical parameters, we assigned CF probands into subgroups based on the categorical attributes listed above and related the cell abundance scores to each subgroup. For both the PBMC and plasma models, fewer monocytes and macrophages were seen in the severe CF subgroups (classes I/II/III and pancreatic insufficient) than in the mild/moderate subgroups (class IV and pancreatic sufficient) or HCs (Figure 5, A–D). *P. aeruginosa* infection was negatively associated with monocyte abundance in the plasma model (Figure 5E) but not in the PBMC model (data not shown). Moreover, in the PBMC model, the abundance of mononuclear phagocyte subsets (monocytes and activated DCs) was negatively correlated with FEV_1_ values while macrophage scores were positively correlated with sweat chloride levels (Figure 5, F and G). Collectively, these analyses indicate that the immune dysregulation associated with CFTR mutations likely results from the loss of innate immune cells and that the deficiency of mononuclear phagocytic cells, in particular, is closely linked to *P. aeruginosa* infection, clinical severity, and the progression of lung function impairment in CF.

### CF trio plasma triggers a robust response in THP-1 monocytes but not macrophages.

**Figure 6:**
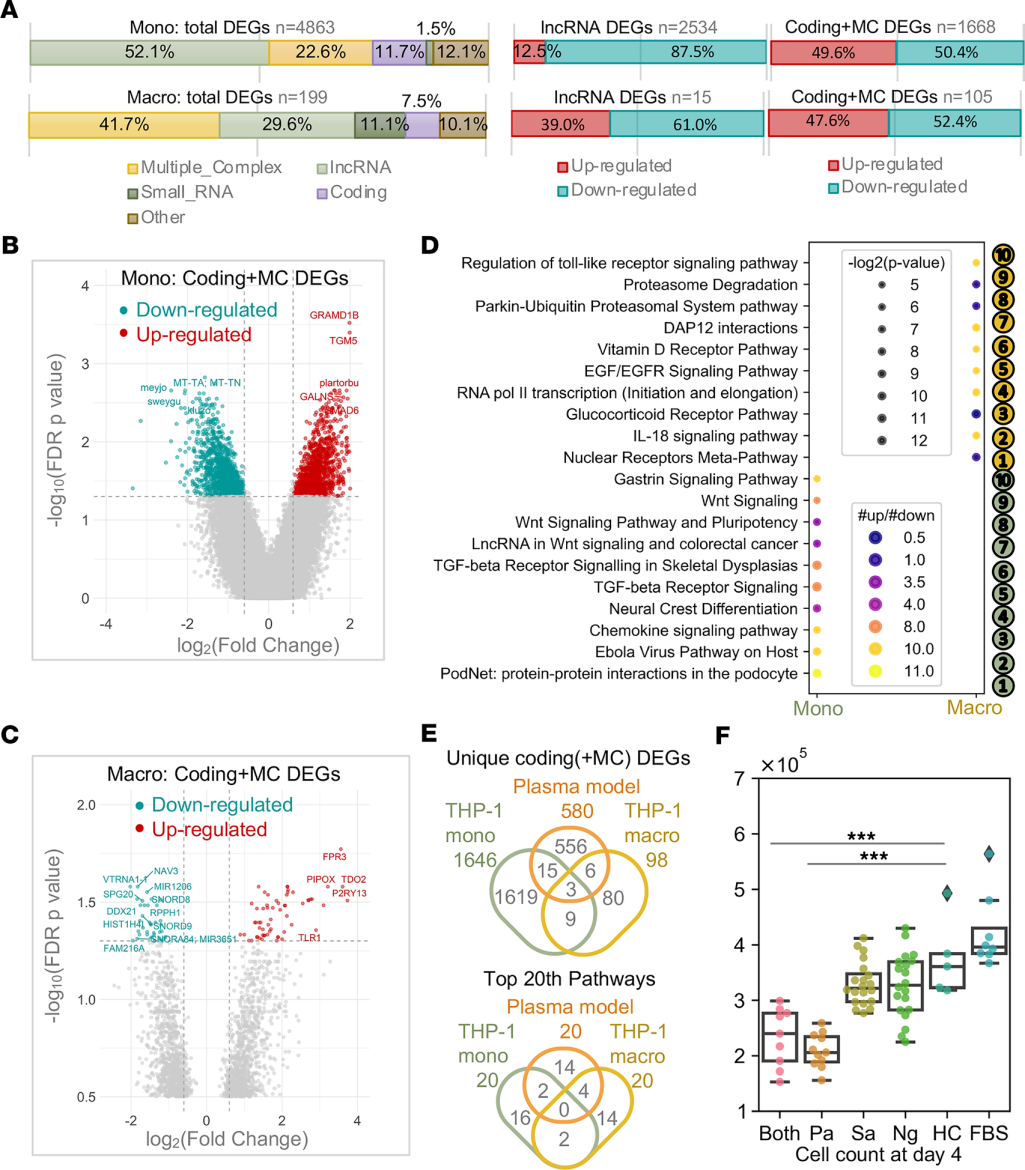
Plasma-cultured monocytes, but not macrophages, show dramatic changes in gene expression. (**A**) Breakdown of differentially expressed transcripts (fold change <–2 or >2, FDR *P* < 0.05, CF probands versus HCs in the THP-1 monocyte and macrophage models) in main categories according to locus type. (**B** and **C**) Volcano plots of (**B**) all differentially expressed transcripts and the (**C**) differentially expressed transcripts in “coding” and “multiple complex” (MC) categories. (**D**) Bubble plot of top 10 significant pathways in WikiPathways ranked by number of regulated genes. (**E**) Venn diagrams showing the numbers and overlap of unique genes (upper) and top 20 pathways (lower) for CF probands versus HCs in the indicated models. (**F**) Bar plot of THP-1 cell numbers after 4 days of culture with CF plasma. Dunn’s multiple comparison for nonparametric post hoc testing was performed following the Kruskal-Wallis test to compare the differences between HC and other groups. ***P < 0.001. *Pa*, *P*. *aeruginosa;* *Sa*, *Staphylococcus aureus*; Ng, negative; FBS, fetal bovine serum; DEGs, differentially expressed genes.

The monocyte cell line THP-1 has been used extensively to study monocyte and macrophage functions, mechanisms, and signaling pathways (34). Given the identification of monocytes and macrophages in the analyses reported above, we focused on a model in which we cultured CF proband plasma with THP-1 cells in the presence or absence of PMA to differentiate these cells into macrophages (Figure 1 and Table 1). Transcriptomic profiling identified more than 4800 DEGs (out of 135,750 transcripts analyzed) in THP-1 monocytic cells cultured with plasma from CF probands or HCs, but only 199 DEGs from THP-1 macrophage-differentiated cells cultured in the same manner (Figure 6A). DEGs from the THP-1 monocytes showed a significantly higher fold change than DEGs from the THP-1 macrophages (Figure 6, B and C). In contrast to the results described above, in which most DEGs in CF proband cells were downregulated in PBMCs (Figure 2, B and C), similar numbers of DEGs were both up- and downregulated in the THP-1 monocytes and macrophages (Figure 6, B and C, and Supplemental Tables 1 and 2). Pathway analysis of DEGs from the THP-1 model (both monocytes and macrophages) revealed enriched immunoregulatory pathways such as transcription factors, cytokines, and receptors (Figure 6D and Supplemental Table 3). Very few genes or pathway signatures were shared between the THP-1, plasma (Figure 6E), and/or the PBMC models (data not shown); however, the THP-1 model specifically represented monocytes (34). In cell growth assays of undifferentiated THP-1 cultures, plasma from CF probands with *P. aeruginosa* infection significantly inhibited the proliferation of these monocytes (Figure 6F). Thus, plasma from CF probands appears to act as a modulator in an immunoregulatory capacity, inducing a broad and strong activation of THP-1 monocytes that is much more robust than that detected in THP-1 macrophages.

**Figure 7:**
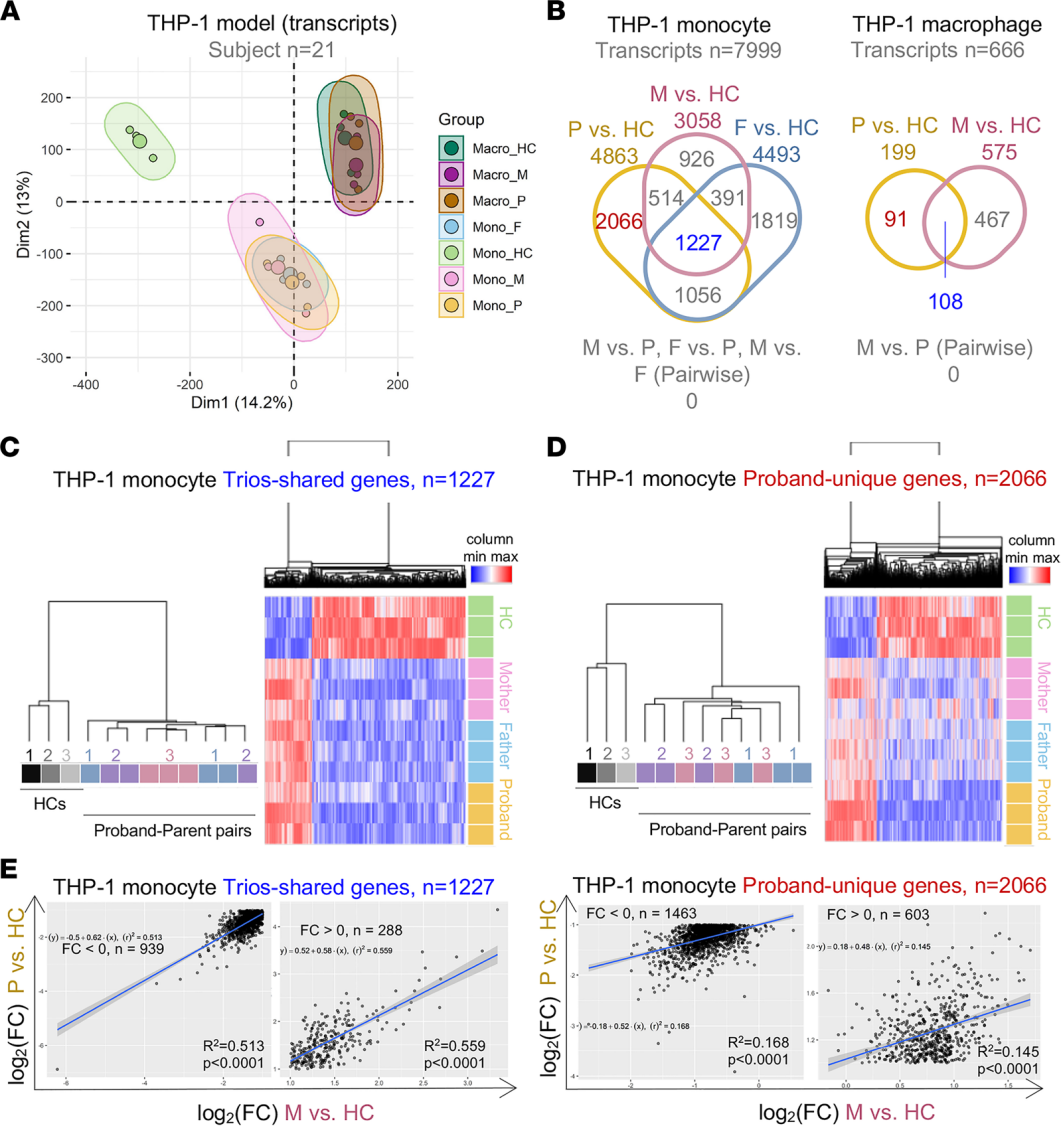
Transcriptomic profiles of CF carriers and probands are less correlated in the THP-1 monocyte model than the PBMC model. (**A**) PCA of THP-1 monocytes and macrophages incubated with study participant plasma based on similarities in transcriptomic profiling. (**B**) Venn diagrams showing the numbers and overlap of DEGs from THP-1 models. (**C** and **D**) Trio-shared and proband-unique genes are highlighted in blue and red, respectively. Hierarchical clustering by trio subgroups and heatmap of gene expression of (**C**) trio-shared genes and (**D**) proband-unique genes from the THP-1 monocyte model. (**E**) Correlation scatterplots of fold changes (log_2_) of the indicated comparison of the fold change (log_2_) of expression of trio-shared (left) and proband-unique genes (right) in the THP-1 model. The *P* value and *R*^2^ (square of the correlation coefficient) were produced by a Pearson’s correlation analysis. The linear regression line and its equation were generated from a simple linear regression analysis. Mono, monocyte; Macro, macrophage; P, proband; F, father; M, mother; FC, fold change.

Next, we determined whether the plasma of CF probands and of their parents evoked similar transcriptomic results in the THP-1 model. PCA of transcriptomic profiling data from both THP-1 monocyte and macrophage samples (*n* = 21 in 7 groups) revealed that monocytes, but not macrophages, from CF trio subgroup clusters always overlapped, whereas the HC clusters remained distinct (Figure 7A). In THP-1 monocytes and macrophages, we identified trio-shared genes (*n* = 1227 and 108, respectively) and proband-unique genes (*n* = 2066 and 91, respectively; see Figure 7B and Supplemental Tables 4 and 5), like the PBMC model (Figure 3B). As in the PBMC model, approximately one-fourth (1227 out of 4863) of the DEGs identified by comparing THP-1 monocytes incubated with CF proband plasma with those incubated with HC plasma were also differentially expressed in THP-1 monocytes incubated with CF parent plasma compared with those incubated with HC plasma (Figure 7B). Trio-shared and proband-unique genes in the THP-1 monocyte model were similarly identified from the total DEGs and used to identify homogeneous groups of participants through hierarchical clustering (Figure 7, C and D). Our analyses confirmed that trio-shared genes reflected familial structures and the expression patterns shared between THP-1 monocytes incubated with CF proband or parent plasma. Moreover, the expression of trio-shared genes showed a significant (*P* < 0.0001) and moderately linear relationship (upregulated: *R*^2^ = 0.513, downregulated: *R*^2^ = 0.559) in THP-1 monocytes incubated with CF proband or parent plasma, whereas proband-unique genes showed weaker correlations in expression between THP-1 monocytes incubated with CF proband or parent plasma (upregulated: *R*^2^ = 0.168, downregulated: *R*^2^ = 0.145) (Figure 7D and Supplemental Figure 6). When we profiled miRNAs using an independent microarray (see Methods), we observed similar miRNA expression patterns across CF trio groups (Supplemental Figure 7A), and miRNA profiling revealed no significant differences in the expression of genes or miRNAs between THP-1 monocytes incubated with CF proband or parent plasma (Supplemental Figure 7B). Therefore, we concluded that plasma from CF parents induces an immune response very similar to that induced by plasma from CF probands.

### Endotoxin tolerance is involved in the immune response CF trio subgroups share.

**Figure 8:**
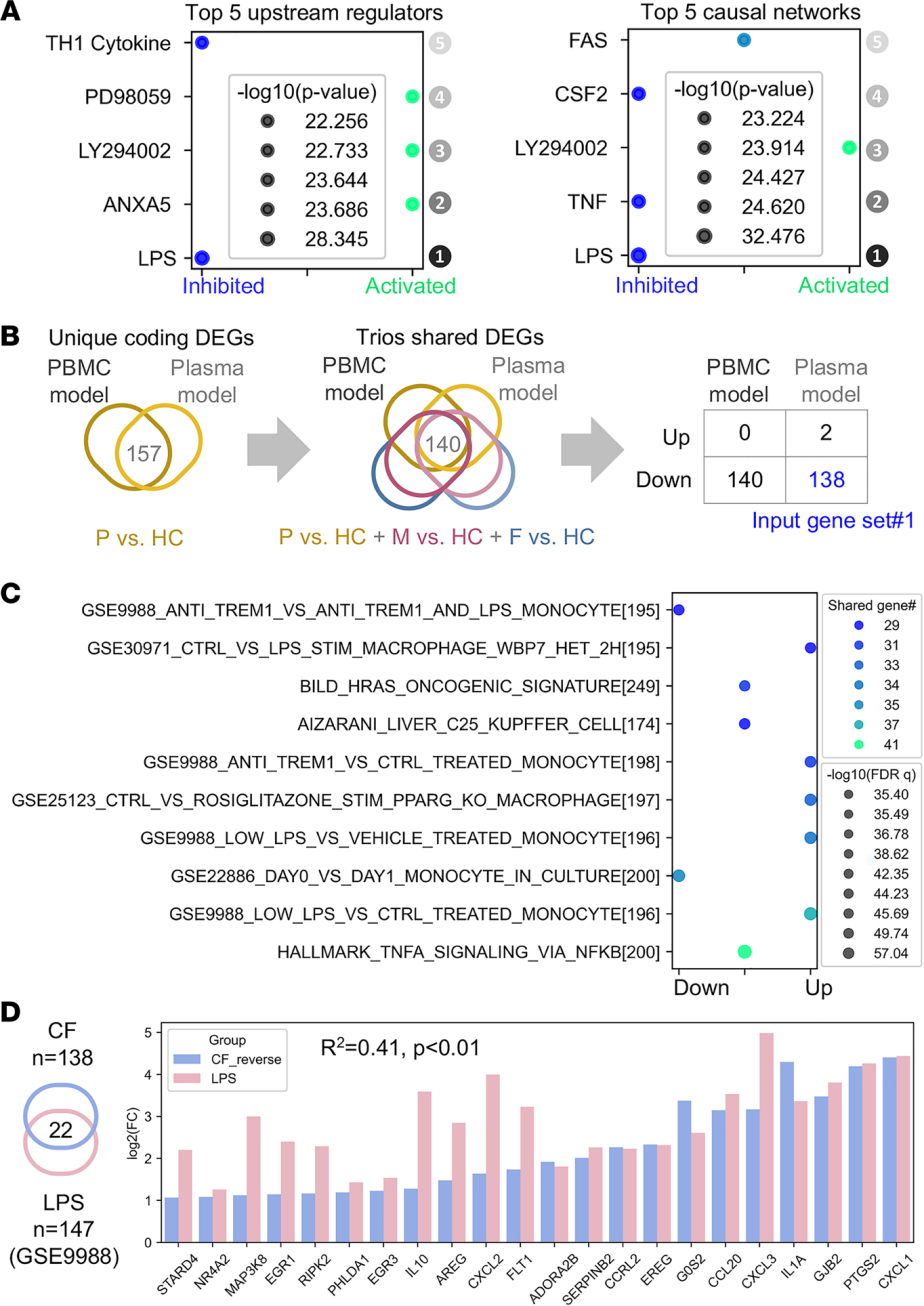
Integrated pathway enrichment analysis suggests an LPS-tolerant state in CF trios. (**A**) Bubble plot of the top 5 significant upstream regulators and causal networks, ranked by *P* value, from Ingenuity Pathway Analysis (IPA) using genetic profiles from the PBMC model (see Methods). (**B**) Flow of identification and selection to identify input gene set 1 for gene set enrichment analysis (GSEA). First, the overlapping coding genes (*n* = 157) from the PBMC and plasma models (CF proband versus HC) were identified. Then, the overlapping genes (*n* = 140) from these genes with DEGs from comparison of CF participants versus HCs were identified; the final input gene set (*n* = 138) were identified as the genes regulated in same directions in both PBMC and plasma models. P, proband; F, father; M, mother. (**C**) Bubble plot of gene sets from GSEA matched with input gene set 1. The top 10 matched gene sets were ranked by *q* value (FDR). (**D**) Left, Venn diagram of the 22 overlapping genes from input gene set 1 and the annotated LPS-inducible gene set (GSE9988). Right, bar plot of fold change (log_2_) of the expression levels of genes in both input gene set 1 and the annotated LPS-inducible gene set (CF or LPS versus HC). Fold change values from input gene set 1 were reversed from negative to positive for ease of visualization. The *P* value and *R*^2^ (square of the correlation coefficient) were produced by a Pearson’s correlation analysis.

Given the similarities between results obtained with PBMCs and plasma from CF probands and parents, we characterized the top-ranked upstream regulators and causal molecular networks in CF probands versus HCs (PBMC model) using Ingenuity Pathway Analysis (IPA; see Methods). Interestingly, we identified LPS, an integral component of the *P. aeruginosa* cell envelope, as the top-ranked upstream regulatory molecule and LPS-associated signaling as the top-ranked causal network inhibited in PBMCs with a *CFTR* mutation (Figure 8A and Supplemental Table 6). To validate this finding using large collections of published studies, we identified 2 core sets of protein-coding genes in the DEGs from the PBMC and plasma models (*n* = 140) and the THP-1 monocyte model (*n* = 365; Figure 8B and Supplemental Figure 8A). Three input gene sets (Supplemental Table 7) that included genes regulated in the same direction (up- or downregulated) were submitted for gene set enrichment analysis (GSEA) in the Molecular Signatures Database (MSigDB) (35–38). Searches on input gene sets 1 and 2 returned significant matches that included gene sets previously defined from PBMCs, monocytes, or macrophages (Figure 8C and Supplemental Figure 8B). Out of the top 10 gene sets that matched input gene set 1, 5 were associated with LPS or TLR4-interacting protein-triggering receptors expressed on myeloid cells-1 (TREM1; Figure 8C). Similarly, input gene set 2 matched with LPS-stimulated gene sets (Supplemental Figure 8B).

We next compared the fold changes in the expression of the LPS- and TREM1-induced genes, which we shared in our profiling result alongside a published data set from the search result (National Center for Biotechnology Information Gene Expression Omnibus [GEO] GSE9988, CF proband versus HCs, THP-1 cells) (39). We observed a significant but modest correlation between genes identified in our experiment and the annotated LPS-induced genes (*R* = 0.41, *P* < 0.01). We did not see such a correlation with the TREM1-induced genes (*R* = 0.001, *P* = 0.88; see Figure 8D and Supplemental Figure 8C). Together, these results suggest that monocytes from CF probands and carriers are less responsive to LPS, implying development of an LPS-tolerant state.

## Discussion

Recent work in our laboratory and others’ work suggest a state of immune dysfunction in CF (4, 8). However, it remains unclear whether this dysfunction arises from a primary intrinsic abnormality in the immune cells or if the dysfunction is a byproduct of the infection microenvironment created by the *CFTR* defect. Abnormal *CFTR* function impairs host defense, mucociliary clearance, and microbicidal activity in the airways; moreover, dysfunctional immune cells contribute to an impaired response to infection (4). Consequently, most patients with CF have intermittent infections with *P. aeruginosa* that can progress to chronic infection (40). We previously demonstrated that patients with CF exhibit changes in inflammation-related transcripts that correlate with disease status (6, 41). Our group (8, 9) and others (18) have identified monocyte and macrophage functions that are impaired in CF, and these functions are not corrected by CFTR modulators.

In the present study, we used 3 cellular models (Figure 1 and Table 1) to capture the impact of the intrinsic deficiency (*CFTR* mutation) on circulating immune cells as well as the impact of the unhealthy immune microenvironment and compromised inflammatory milieu on circulating immune cells—particularly monocytes and macrophages—in CF probands and carrier parents. Our genomic and transcriptomic analyses identified DEGs that were associated with decreased activity in CF PBMCs. Our analyses also revealed changes in the abundance of mononuclear phagocytes (monocytes, macrophages, and DCs) associated with CF severity, *P. aeruginosa* infection status, and disease progression. In addition, our THP-1 model demonstrated that monocytes — rather than monocyte-derived macrophages — responded dramatically to CF proband or carrier parent plasma. GSEA identified downregulation of LPS signaling in all cellular models, suggesting a loss of reactivity to LPS in CF PBMCs and monocytes.

Most existing CF profiling studies (42–46) have concentrated on CF airways and lungs, but CF immune dysfunction is likely extrapulmonic, involving systemic alteration of immune function allowing for persistence of chronic infection (4, 11, 47). Consistent with this hypothesis, the immune-related transcriptomic profiles identified here (Figure 2, A–D) suggest that the innate immunodeficiency in CF is apparent outside of the chronically infected environment of the lung, a deficiency also seen in CF carrier parents.

In all 3 cellular models, we uncovered no significant differences between transcriptomic profiles or those the plasma of CF probands and carrier parents induced (Figure 3B and Figure 7B). We did, however, identify groups of DEGs that were shared and coexpressed in patients with CF and carrier parents (trio-shared genes) versus HCs (Figure 3, C and E, and Figure 7C). These results are not surprising given prior evidence suggesting that a genetic load of 50% wild-type *CFTR* is not sufficient for maintaining health (24, 27). CF carriers may constitute a haploinsufficient population that is at a higher risk than the noncarrier population for developing respiratory infections and other diseases commonly associated with CF (23, 25–27).

PBMCs are a diverse mixture of highly specialized immune cell subsets that include myeloid and lymphoid cells (48). Under physiological conditions in healthy people, CFTR protein is abundantly expressed in airway epithelial cells but expressed at lower levels in PBMCs (49). Nonetheless, CFTR is believed to carry out an irreplaceable function in myeloid cells (11, 18, 50). Notably, Sun and colleagues identified a set of genes in PBMCs that predict clinical responsiveness to ivacaftor therapy. Using IPA, they mapped these genes to cellular processes that regulate innate immunity and inflammation (51). It remains unclear, however, whether these transcriptomic alterations were a direct effect of CFTR modulation in PBMCs or instead reflected systemic effects due to correction of the pathological environment generated from the *CFTR* defect. Employing processes used to identify genes associated with ivacaftor responsiveness (51), we found that unlike HC PBMCs and plasma-cultured counterparts, the innate immune pathway was downregulated in *CFTR*-mutated PBMCs and in CF plasma-cultured healthy PBMCs and THP-1 monocytes (Figure 2D and Figure 6D).

Monocytes typically account for 10%–20% of total PBMCs found in blood (48), and these circulating monocytes are highly phagocytic (52, 53). Several powerful in silico approaches have been established to monitor changes in immune cell composition, using transcriptomic profiles to reveal distinct functionalities in cell subsets (54, 55). Our observations that macrophages are significantly less abundant in CF trio subgroups than in HCs (Figure 4B) are consistent with the findings of our previous study (8) as well as a recent report that the intrinsic molecular mechanisms controlling leukocyte recruitment and migration are severely impaired in CF monocytes (56). Our observation that monocytes showed greater differences in DEGs than macrophages upon treatment with CF plasma may result from activated cells being less responsive to CF plasma. Taken together, these results support the notion that *CFTR* mutations lead to immune dysfunction and deficiency. Notably, ivacaftor treatment does not change the abundance of PBMCs or the composition of immune subsets (monocytes, T cells, or B cells) (57). Recently, it was shown that CRISPR/Cas9-mediated knockout of CFTR in human macrophages results in decreased phagocytosis and increased bacterial load (58), supporting our finding that innate immune cell dysfunction is CFTR dependent. Further, these findings suggest that the cumulative abnormality in CF innate immunity is primarily caused by the fluid microenvironment resulting from the consequences of defective *CFTR* function. Since the intrinsic *CFTR* mutation disrupts monocyte recruitment and migration and cytokine levels and cell-to-cell interactions play major roles in this regulation (59), it follows that CFTR modulator therapy would not effectively reverse these consequences (60).

Given that individuals with CF commonly experience lung disease progression caused by chronic *P. aeruginosa* colonization (14, 15), it is not surprising that *P. aeruginosa* infection was associated with monocyte/macrophage abundance (Figure 5E), as observed in our previous study (8). We also found evidence of negative associations between monocyte/macrophage abundance and CF disease severity (Figure 5, A–D, F, and G). Interestingly, these associations were not equally represented in the plasma and PBMC models: changes in monocyte/macrophage abundance were more strongly associated with *CFTR* mutation class (Figure 5A) and pancreatic status (Figure 5B) in the plasma model but more strongly associated with sweat chloride and FEV_1_ levels in the PBMC model (Figure 5, F and G). These findings add to a growing body of evidence that the abundance of circulating monocytes can predict disease severity and progression as seen in chronic obstructive pulmonary disease (61, 62) and idiopathic pulmonary fibrosis (63).

In our quest to identify the genes and pathways that underlie impaired innate immunity in CF, we uncovered a set of gene signatures (input gene set 1) that was shared by CF trio subgroups relative to HCs and downregulated in both plasma and PBMC models (Figure 8B). The gene signatures identified in the PBMC model (Figure 3C) better represented the features shared by CF trios than signatures identified from THP-1 cells (Figure 7C) because the correlation of the transcriptomic profiles between CF probands and carrier parents was much stronger in PBMCs (Figure 3E) than in THP-1 cells (Figure 7E). Further, GSEA revealed that these signature genes highly overlapped with LPS- and TREM1-induced genes, although these genes were regulated in opposite directions (Figure 8, C and D). The significance of LPS or TREM1 as upstream regulators was supported by an independent IPA performed on data from the PBMC model (Figure 8A) and previous analyses of the plasma model (6, 8). As an activating receptor expressed on monocytes, TREM1 interacts and synergizes with the LPS/TLR4 receptor complex to trigger a respiratory burst, phagocytosis, and cytokine release in the innate immune system (39, 64, 65). However, CF monocytes are locked in an endotoxin-tolerant state (66) that is at least partly due to robust downregulation of TREM1 (67). Research suggests that a soluble endotoxin present in the bloodstream of individuals with CF may cause endotoxin tolerance in circulating monocytes (68). Consistent with these reports, we detected lower expression of LPS- and TREM1-induced genes (Figure 8C), suggesting that CF PBMCs are less responsive to LPS and have acquired a tolerant state. However, the presence of soluble endotoxin has not yet been independently verified, and the downregulation of TREM1 in patients with CF has recently been challenged (69). Given that patients with CF can develop chronic *P. aeruginosa* infection (70), we wonder how long the tolerance to LPS can last and whether the tolerance is reversible. Better understanding of longitudinal exposure to LPS in patients with CF may yield important mechanistic insights into the causes and consequences of LPS tolerance.

Although supportive of the concept of immune dysfunction and immunodeficiency, the present study includes some limitations. First, we identified several differentially expressed lncRNAs (Figure 2A and Figure 6A) in both the PBMC and THP-1 models. However, the clinical significance of these lncRNAs remains unclear, partly due to a lack of detailed information about their biological functions. Although a significant number of lncRNAs have been implicated in LPS tolerance (71, 72), it remains unknown how lncRNAs suppress the LPS- and TREM1-induced pathways. A longitudinal study is needed to highlight lncRNAs with therapeutic potential for CF. Second, due to the small sample size in the PBMC model (Table 1) and the heterogeneity of human participants, transcriptomic profiling failed to reveal transcripts that were significantly differentially expressed (Figure 3B and Figure 7B) or differentially spliced (data not shown) between a proband with CF and either of the parents. Third, we initially did not adjust for multiple-hypothesis testing when we ran a correlation analysis between cell abundance and indicators of disease severity and progression (sweat chloride and FEV_1_ levels; Figure 5G). While a Bonferroni’s correction can reduce the chance of false positive findings, this correction is overly conservative when statistical tests are correlated, as is the case for immune cell composition scores. However, when comparing all cell type scores between groups, we adjusted for the number of pairwise comparisons within each score, such as proband versus mother and proband versus HC, but we did not adjust for the number of immune cell scores. Within each immune profile score, *P* values were adjusted for multiple comparisons using Holm-Šidák method to control the family-wise error rate. The Holm-Šidák method is uniformly more powerful than a Bonferroni’s correction while maintaining strong control of family-wise type 1 error rates. The study did not address the potential influence of differences in lung microbiota on the responses. Last, although THP-1 cells were introduced as an alternative option rather than primary PBMCs to validate the PBMC model, they are not equal to the status of primary cells.

While homozygous mutations in *CFTR* cause CF, several studies suggest that heterozygous *CFTR* mutations have functional consequences (23). Our study has revealed similarity in the effects of *CFTR* mutation on innate immune cell populations in CF probands and carrier parents. CF carriers have an increased risk of developing airway obstruction (23, 73), neutrophil abnormalities, and ineffectual macrophage apoptosis (74). This study adds to the growing literature suggesting that the effects of heterozygous *CFTR* mutations on circulating monocytes could increase the prevalence of infection, ultimately leading to chronic bronchitis and severe lung disease (8, 75). The LPS-tolerant phenotype detected in this study may help explain the susceptibility present in carriers of homozygous and heterozygous *CFTR* mutations. Increasing our understanding of the role of novel lncRNAs in mediating signaling will pave a path for improved, targeted therapies as new research begins uncovering the role of lncRNAs as master regulators of gene expression.

## Methods

### Study participants and data collection.

A total of 164 CF trio participants (CF probands, fathers, and mothers) from 100 CF families and 20 unrelated HC participants were recruited from the Children’s Hospital of Wisconsin (CHW 07/72, GC 390, CTSI 847, CHW 01-15), Ann & Robert H. Lurie Children’s Hospital of Chicago (2015-400), and National Jewish Health (NJH HS-3648). This study (Figure 1) was approved by the Institutional Review Board (IRB) at each institution after scientific and ethical review. The Biomedical Research Alliance of New York (BRANY) is the IRB providing current oversight at National Jewish Health. HC participants were free of known infection at the time of sample collection. Additional disease-free HC samples (*n* = 11) were obtained commercially (Cellular Technology Limited). Informed consent was obtained from participants or their parents or legal guardians. As described in our prior studies (6, 8), CF proband participants were diagnosed based on pilocarpine iontophoresis (CF Foundation guidelines) (76), symptoms, pancreatic status, *CFTR* mutation class, family history of CF, and information about the phenotypes of *CFTR* mutations (74, 77–79) (details in Supplemental Methods).

General demographic information, such as age, sex, and genotype, was collected through standardized questionnaires. Pancreatic sufficiency status was defined based on levels of fecal pancreatic elastase, with a threshold of 200 mcg/g for sufficiency (74). *P. aeruginosa* infection data were collected during standard screening for microbiological flora, in which the infection was reported as 1 positive microbiological growth from nasopharyngeal, sputum, or bronchoalveolar lavage specimens within 6 months of study enrollment. FEV_1_ data were collected during clinical lung function measurements performed at baseline according to ATS/ERS Task Force guidelines (80). Sweat chloride values were collected based on the sweat tests performed closest to the date of serum sample collection.

### Sample collection and cellular models.

Human PBMCs or plasma samples from CF probands, siblings, parents, and unrelated HCs were aseptically collected in acid citrate dextrose solution A or K^+^ EDTA anticoagulant for the 3 cellular models for molecular profiling (Figure 1 and Table 1). For the PBMC model, PBMCs (buffy coat) were collected from whole blood by Ficoll Paque (GE Healthcare, now Cytiva) density centrifugation at 1800*g* for 15 minutes at room temperature. The PBMCs were then stored frozen in a cryoprotective medium containing 10% dimethyl sulfoxide and 90% FBS. Cryopreserved PBMCs were thawed quickly before RNA isolation or live-cell recovery. The procedure used to develop the plasma model has been described in our prior studies (6, 8, 9). Briefly, healthy human PBMCs (UPN727; Cellular Technology Limited) were cocultured with the plasma collected from the enrolled participants for 9 hours prior to sample collection for transcriptomic analyses. The THP-1 cell line was a gift from Peter H. Sporn (Northwestern University, Evanston, Illinois, USA; originally obtained from ATCC) and was maintained in RPMI 1640 medium (Thermo Fisher Scientific) supplemented with 10% FBS and 2 mmol/L l-glutamine. THP-1 monocytes were differentiated into macrophages by adding 200 nM PMA (MilliporeSigma) to the media for 48 hours. The media were then removed, and THP-1 monocytes or macrophages were cocultured for 9 hours with plasma (without PMA) collected from the enrolled participants.

Table 1 shows the numbers of CF trios and HC participants used to develop the 3 cellular models. In addition to molecular profiling, cell samples from both the PBMC and THP-1 models were examined by cell growth assays or fluorescence-activated cell sorting (Figure 1 and Supplemental Methods). For cell growth assays, THP-1 monocytes with no PMA treatment were cultured in media supplemented with FBS (n = 3) and plasma from HC participants (*n* = 5) or CF probands who tested positive for *P. aeruginosa* (*n* = 13), positive for *Staphylococcus aureus* (*n* = 22), positive for both (*n* = 9), or negative for both (*n* = 20).

### Molecular profiling and data processing.

Total RNA was isolated using TRIzol (Invitrogen), and the purity and concentration were verified using a NanoDrop ND-1000 instrument (Thermo Fisher Scientific). The integrity of the RNA was assessed by a 2100 Bioanalyzer gel image analysis system (Agilent). At least 300 ng mRNA per sample was submitted for library construction, in which each purified RNA sample was transcribed to double-stranded cDNA followed by cRNA synthesis and biotin labeling. The labeled samples were then hybridized onto 3 arrays (Table 1): GeneChip Human Genome U133 Plus 2.0 array (Thermo Fisher Scientific, >54,000 probes and >38,500 genes), Human Clariom D array (Thermo Fisher Scientific, >6,765,500 probes, >542,500 transcripts, and >134,700 genes), and GeneChip miRNA 3.0 array (Thermo Fisher Scientific, 1105 human miRNAs), as reported previously (6, 7). The profiling data from these arrays were normalized in the robust multiarray average procedure, then processed using Transcriptome Analysis Console (Thermo Fisher Scientific, version 4.0) following the manufacturer’s instructions. Data sets for this investigation have been deposited in the National Center for Biotechnology Information, GEO, and are accessible through accession GSE192523. Any additional data will be submitted to this publicly available resource.

### Immune cell profiling.

Immune cell profiling or cell composition analysis was performed using a signature matrix optimized for human PBMC deconvolution, as reported (8). Briefly, more than 20 candidate marker genes for 10 cell subsets in PBMCs were selected from a previously described matrix based on their expression patterns across immune cell subsets (81). The pairwise similarity statistic of all cell subsets (Supplemental Figures 3 and 4) was computed between all pairs of the candidate marker genes within the normalized gene expression data from the PBMC model. Using the criteria (average Pearson’s correlation factor > 0.50, *P* < 0.01), a number of selected marker genes were identified as our final marker genes (8). The raw cell composition score was calculated as the sum of the simple averages of the marker genes’ log_2_ expression, which allows comparison of cell composition across participant groups and subgroups.

### Statistics.

Bioinformatics and statistical analyses were performed and visualized using R version 3.6.1, Python version 3.7.9, Transcriptome Analysis Console (Thermo Fisher Scientific, version 4.0), Prism 7 (GraphPad), IPA (Qiagen), and GSEA databases (MSigDB version 7.2). Relative microarray gene expression levels were compared between groups using an empirical Bayes method (1-way ANOVA followed by eBayes analysis) to share information across genes and generated an improved estimate for the variance. Coding genes and transcripts that displayed at least a 2-fold difference in gene expression between comparison groups (FDR < 0.05) were considered significantly differentially expressed and carried forward in the analysis. FDR adjustment was performed following Benjamini-Hochberg FDR-controlling procedure (82). Differentially expressed RNAs were illustrated as a volcano plot. Hierarchical clustering was performed to show the gene expression patterns and similarities among samples. PCA was performed to cluster participants based on the differentially expressed transcripts. GSEA was carried out by searching the established MSigDB gene set collections (C1, C2, C7, C8) and utilized the top 10 gene sets that matched with our input gene sets.

Associations between cell composition scores and clinical features were evaluated using Pearson’s correlation, *R* (correlation coefficient), and *R*^2^ (square of the correlation coefficient; Figure 5). Immune profile scores were compared between groups and subgroups; independent 2-tailed *t* tests were used when comparing CF trio samples with HCs, and paired 2-tailed *t* tests were used when comparing CF trio samples, assuming a normal distribution and equal variances. Within each immune profile score, *P* values were adjusted for multiple comparisons using Holm-Šidák method to control the family-wise error rate. We compared the cell growth rates between groups using Dunn’s multiple-comparison correction for nonparametric variables; post hoc testing was performed following the Kruskal-Wallis test. A *P* value of less than 0.05 was considered statistically significant. The box plots in figures depict the minimum level (lower whisker) indicating the minimum value in the data set (excluding outliers, Q1 − 1.5×IQR), the maximum level (upper whisker) indicating the maximum value in the data set (excluding outliers, Q3 + 1.5×IQR), the upper and lower quartiles, and the median. The length of the box represents the interquartile range.

### Study approval.

This study was approved by the IRBs (CHW 07/72, GC 390, CTSI 847, CHW 01-15 Children’s Hospital of Wisconsin; 2015-400 Ann & Robert H. Lurie Children’s Hospital of Chicago; NJH HS-3648 National Jewish Health; Figure 1). BRANY is the IRB providing current oversight at National Jewish Health. Written informed consent was received for each study participant. For all enrollees aged less than 18 years, a parent completed and signed the informed consent document.

## Author contributions

XZ performed the experiments, built the data analysis pipeline, analyzed the data, and wrote the first draft of the manuscript. JD performed experiments, edited the manuscript, and submitted the data to GEO. CMM and JRK assisted with the data analyses; CM performed the multiple-comparison adjustment. CM, LDH, VRR, JEI, KAW, S Jump, S Jia, JWP, RB, MJH, and EWG reviewed the manuscript and provided advice. HL devised the concept of the research incorporating the trio comparisons, supervised and implemented all aspects of the study, reviewed data and the first draft, edited the manuscript, wrote the subsequent drafts, and submitted the revision.

## Supplementary Material

Supplemental data

Supplemental table 1

Supplemental table 2

Supplemental table 3

Supplemental table 4

Supplemental table 5

Supplemental table 6

Supplemental table 7

## Figures and Tables

**Table 1:**
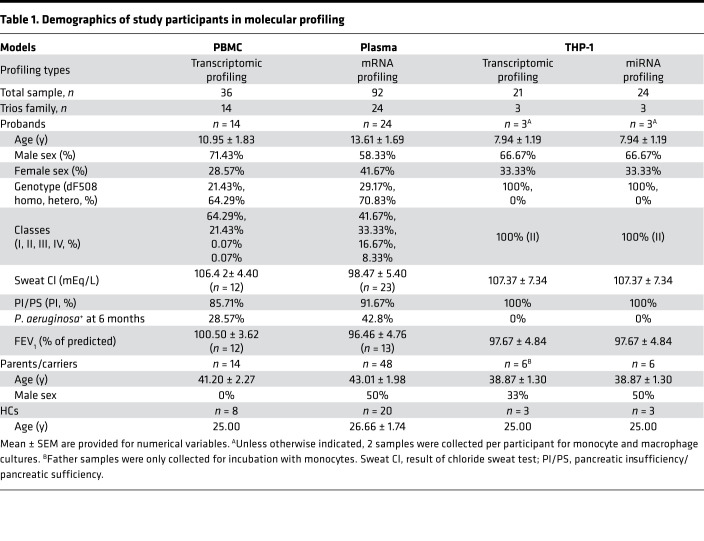
Demographics of study participants in molecular profiling

## Version 1 03/22/2022

Electronic publication
