# Supplementary material for: CFTR-mediated monocyte/macrophage dysfunction revealed by cystic fibrosis proband-parent comparisons
Source: JCI Insight. 2022 Mar 22;7(6):e152186. doi: 10.1172/jci.insight.152186 (PMC8986072; doi:10.1172/jci.insight.152186)
Supplement: Supplemental data [file jciinsight-7-152186-s231.pdf]

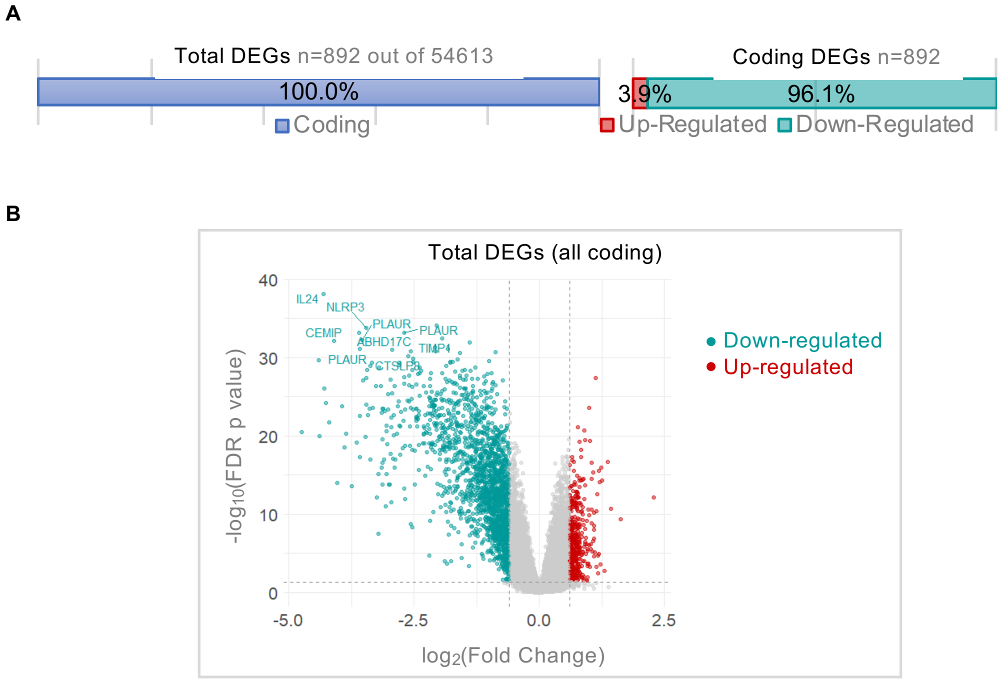

Figure S1. Coding RNAs are significantly downregulated in donor PBMCs cultured with CF plasma. (A) Breakdown of differentially expressed mRNAs (fold change < -2 or > 2, false discovery rate $p < 0.05$, CF proband vs. HCs in the plasma model) in main categories of locus type. **(B)** Volcano plot of total differentially expressed mRNAs.

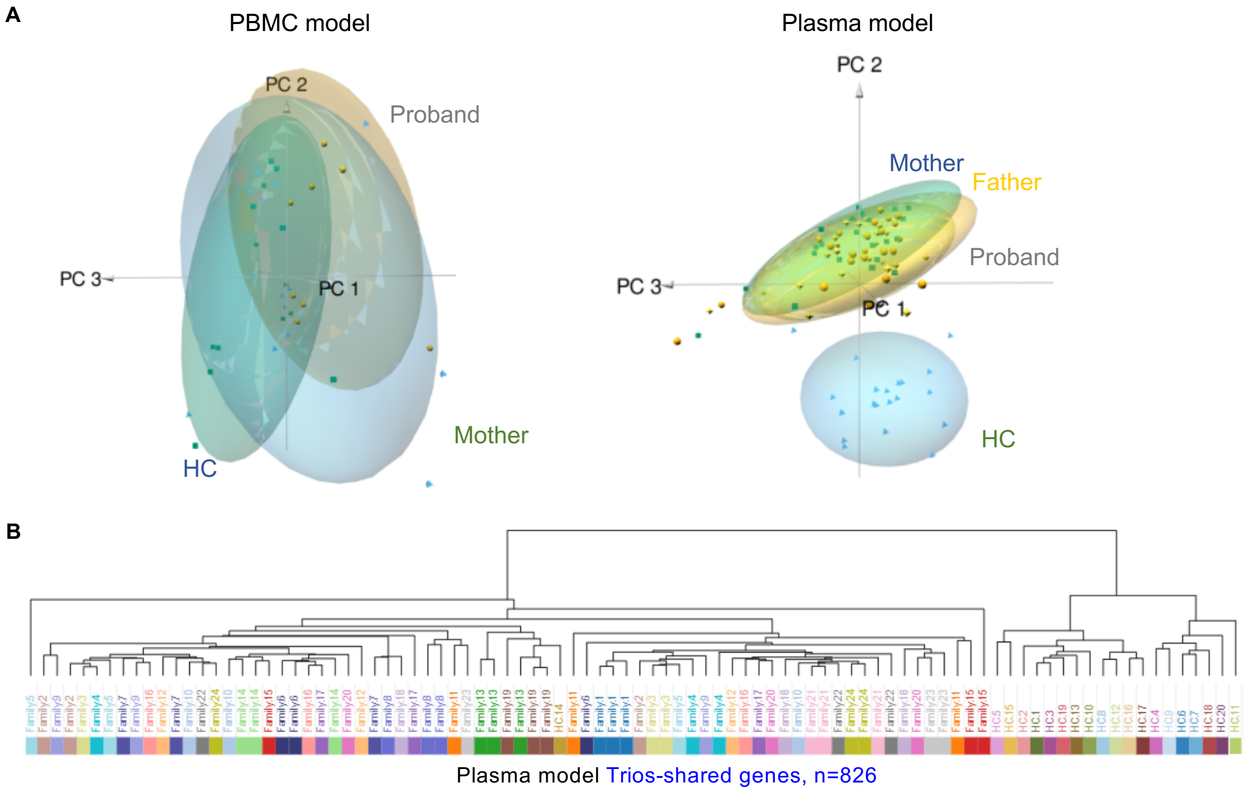

Figure S2. PCA and hierarchical clustering of data from PBMC and plasma models. (A) 3D PCA of clusters of study subjects in the PBMC (left, n=36) and plasma models (right, n=92). **(B)** Hierarchical clustering of study subjects using "trios-shared genes" from the plasma model.

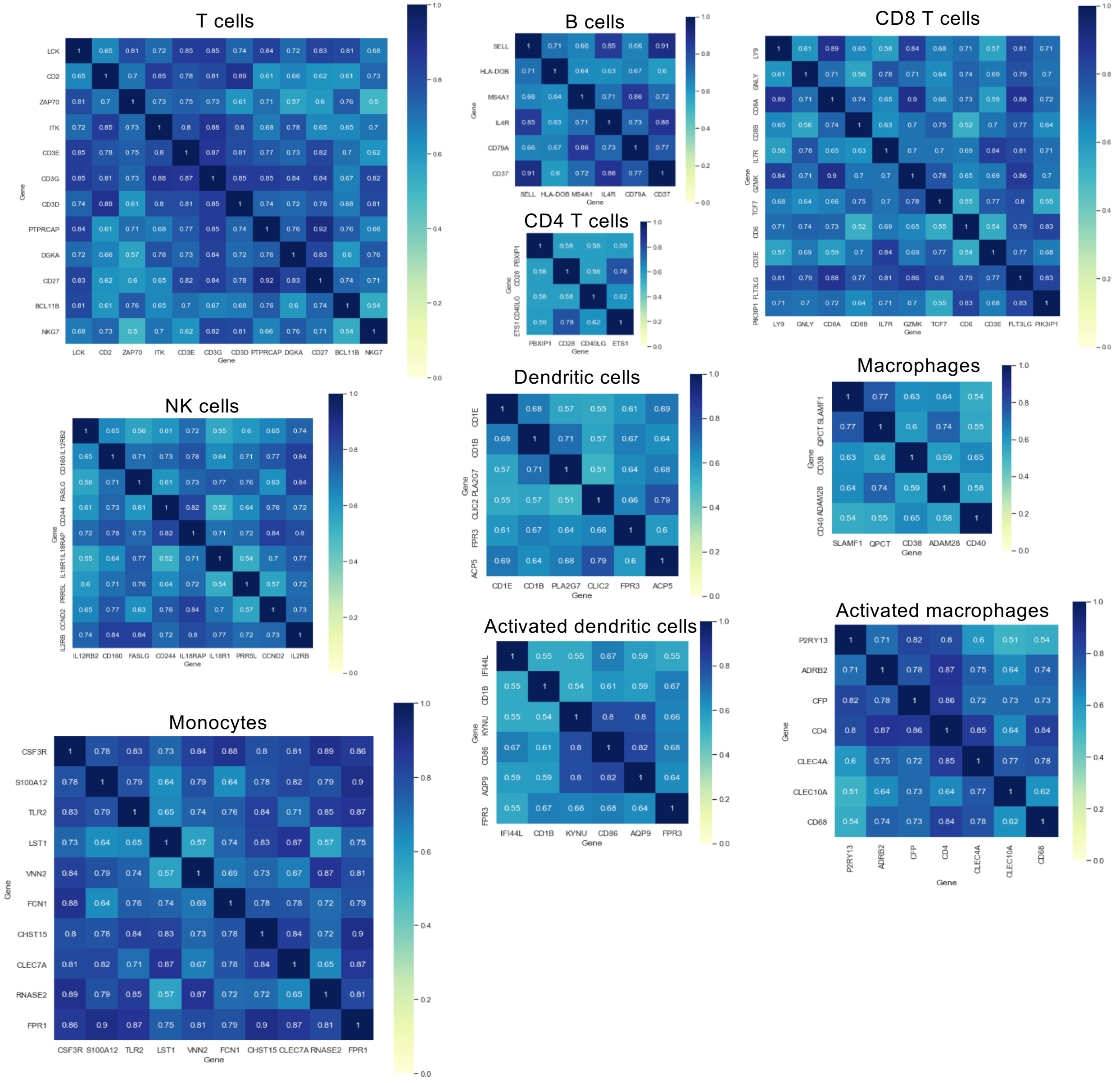

Figure S3. Pairwise similarity of predefined marker genes in ten cell subsets from the PBMC model. Pairwise similarity was computed based on transcriptomic expression across all subjects.

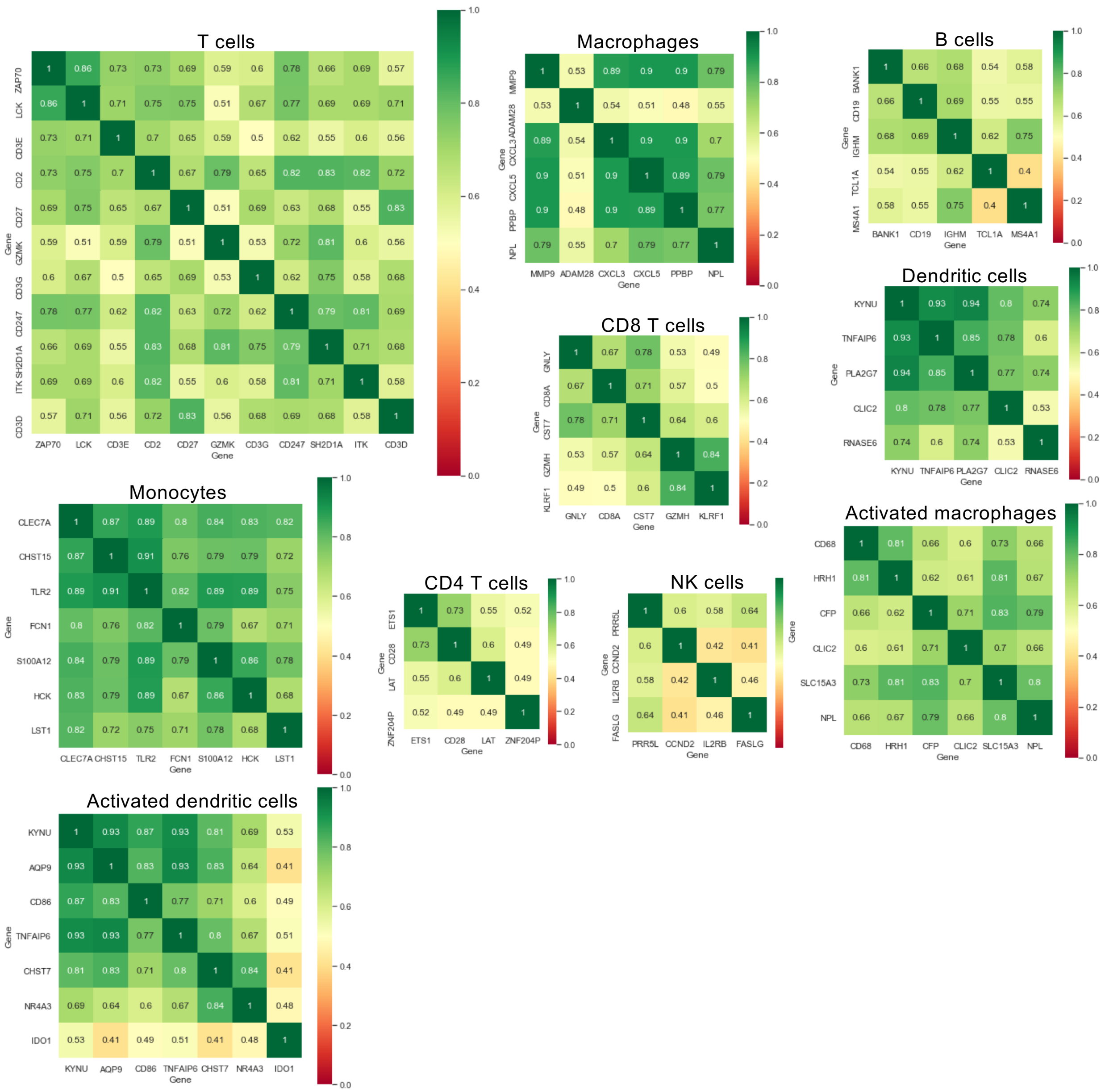

Figure S4. Pairwise similarity of predefined marker genes in ten cell subsets from the plasma model. Pairwise similarity was computed based on transcriptomic expression across all subjects.

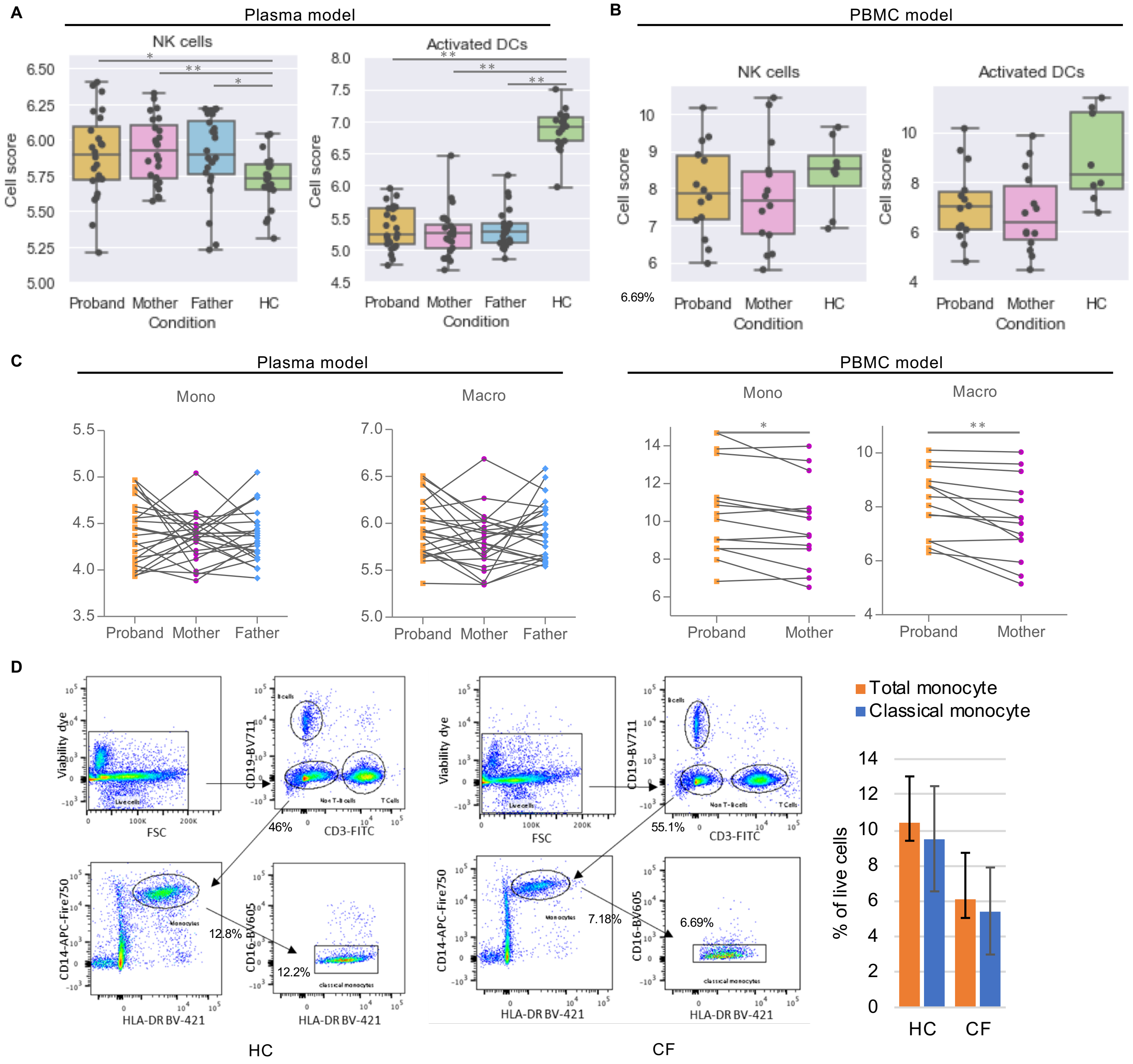

Figure S5. Additional immune-cell composition analysis of CF parent-child trios and HCs. (A-B) Dot plot and box plot of the cell composition scores of two additional cell subsets in **(A)** the plasma model and **(B)** the PBMC model. The equality of variances was tested and confirmed by F-test; the normal distribution in each sample group were tested and confirmed by Shapiro-Wilk normality test; the means of all comparison pair were compared by unpaired independent t-test (equal variances and normal distribution assumed). **(C)** Dot plot of the cell composition scores of monocytes and macrophages in (left) the plasma model and (right) the PBMC model. The means of all comparison pair were compared by paired t-test (two subgroups) or ANOVA (three groups) followed by Turkeys Multiple comparison. **(D)** Flow cytometry (Methods) of circulating total monocytes and classical monocytes in CF probands (left) and HCs (middle). Representative dot plots are shown. Arrows represent gating strategy and flow of selection of specific cell populations. Right, mean values of monocyte percentages of the two groups (HCs, n=3; CF, n=9). *$p < 0.05$, **$p < 0.01$.

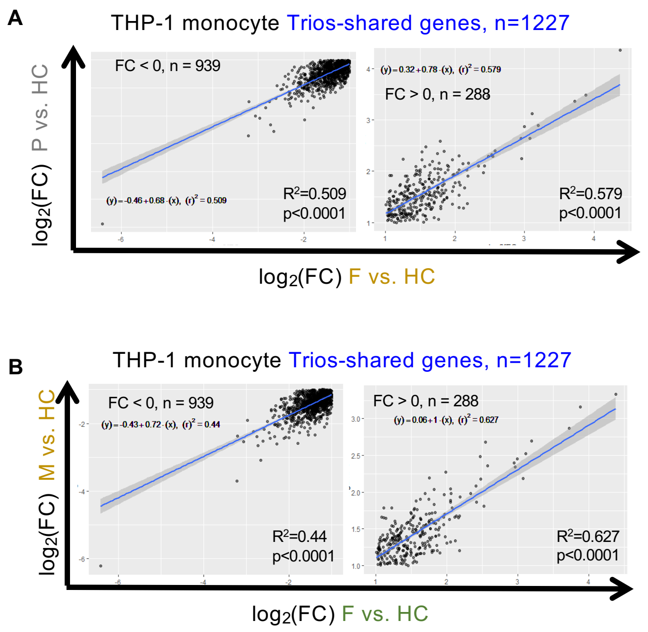

Figure S6. Transcriptomic profiles of CF carriers and probands are moderately correlated in the THP-1 monocyte model. Co-correlation scatter plots of the fold changes (log_2) of the expression levels of "trios-shared genes" in **(A)** proband and father versus HCs and **(B)** mother and father versus HCs in the THP-1 monocyte model. The p value and R^2 squared (square of the correlation coefficient) were produced by a Pearson correlation analysis. The linear regression line and its equation were generated from a simple linear regression analysis. P, proband; M, mother; F, father; FC, fold change.

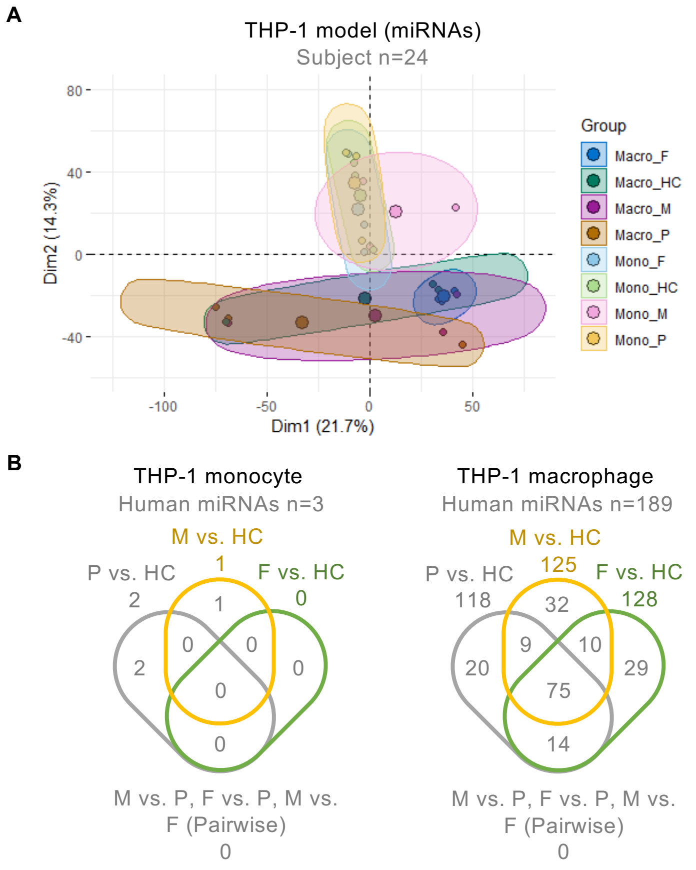

Figure S7. miRNA profiling of CF carriers and probands identifies limited signatures in THP-1 monocytes. (A) PCA of data from study subjects in THP-1 model based on their similarity in an independent miRNA profiling experiment (Methods). **(B)** Venn diagrams of numbers and overlap of miRNA signatures from the THP-1 models. P, proband; M, mother; F, father.

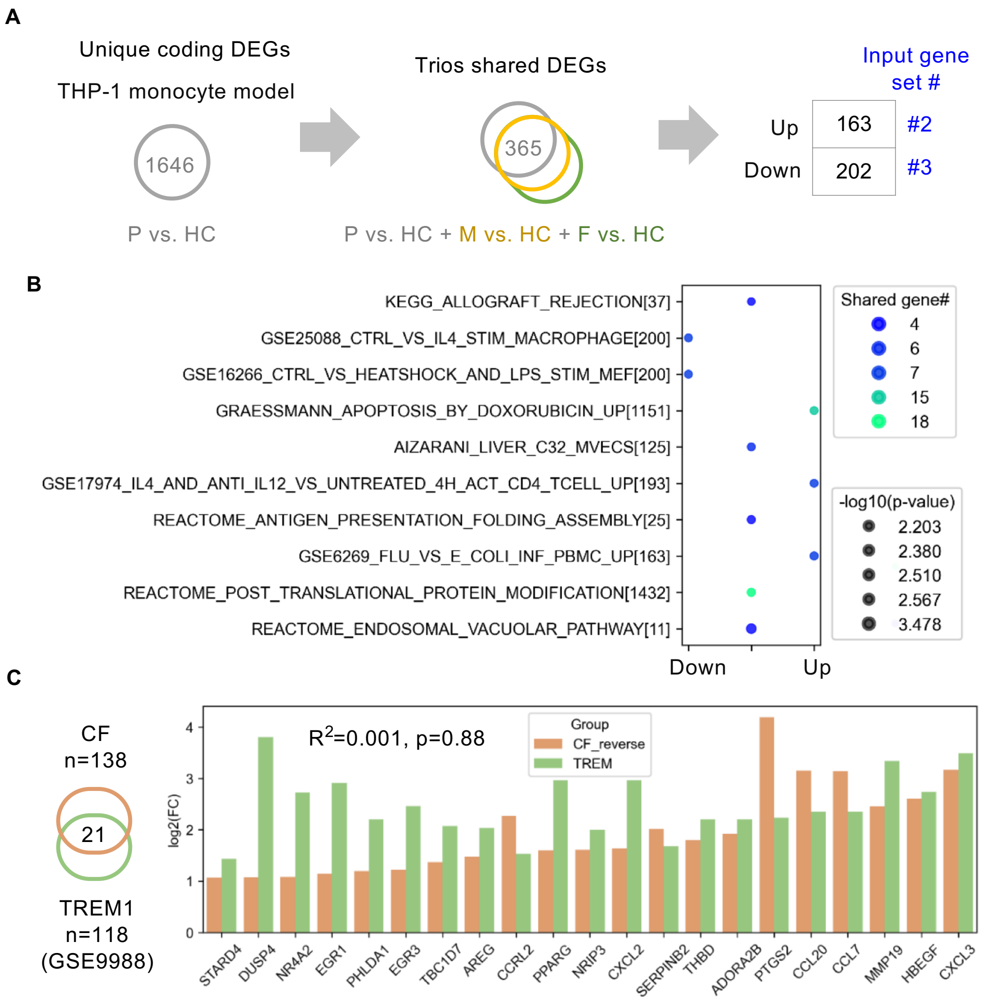

Figure S8. Additional pathway enrichment analysis supports an "LPS tolerant" state in CF trios. (A) Flow of identification and selection to identify input gene set#2 and set#3 for GSEA. **(B)** Bubble plot of gene sets from GSEA matched with input gene set#2. The top 10 matched gene sets were ranked by q-value (false discovery rate). P, proband; M, mother; F, father. **(C)** Left, Venn diagram of the 21 overlapping genes from input gene set#2 and the annotated TREM1-inducible gene set (GSE9988). Right, bar plot of fold change (log_2) of the genes present in both gene sets (CF or TREM-1 versus HC). Fold-change values from input gene set#2 were reversed from negative to positive for the convenience of visualization. The p-value and R^2 (square of the correlation coefficient) were produced by Pearson correlation analysis.

Supplementary Method

Study subject enrollment

The detailed inclusion criteria for CF proband subjects included: 1) a confirmed CF diagnosis (sweat chloride value ≥60 mmol/L and/or CFTR genotype documenting 2 CF-causing mutations); 2) follow-up at least once per year; and 3) CFTR genotyping performed at the time of diagnosis or, in older subjects, after genetic testing became available. Patients diagnosed via newborn screening were evaluated by the Wisconsin Newborn Screening Laboratory for the recommended American College of Medical Genetics panel of 23 CFTR mutations (7). Additional genetic testing was carried out for patients with 1 identified mutation, including expanded mutation panel testing (Genzyme Genetics, Cambridge, MA), modified temporal temperature gradient electrophoresis of CFTR (Ambry Genetics, Aliso Viejo, CA), and multiplex ligation-dependent probe amplification for deletions and duplications (Ambry Genetics).

Flow Cytometry (FACS) analysis

Human PBMCs were purified from whole blood by Ficoll density gradient centrifugation, washed with FACS buffer (5% BSA (Sigma-Aldrich) and 1x PBS) and blocked with Human TruStain FcX (Biolegend) for 20 min. at 4°C. Cells were spun down, resuspended in 100 ul of FACS buffer, and stained for multicolor flow analysis using 2.5 ul of each fluorescently conjugated antibody (specific to the surface markers to be visualized) and 1 ul viability dye per 1 x 10^6 cells for 30 min at 4°C. Cells were then washed 2x with FACS buffer, spun down, resuspended in 100 ul IC Fixation Buffer (Invitrogen) and fixed at RT for 10-15 min. Cell were spun down and resuspended in FACS buffer. Stained PBMCs were then analyzed on the BD LSRFortessa Analyzer. Single cell stains were also processed as described and used as controls for compensation purposes.

All antibodies used for immunophenotyping were purchased from Biolegend and are as follows: Alexa Fluor 700 anti-human CD8, clone SK1; APC anti-human CD4, clone OKT4; APC/Fire 750 anti-human CD14; Brilliant Violet 421 anti-human HLA-DR, clone 63D3; Brilliant Violet 605 anti-human CD16, clone 3G8; Brilliant Violet 711 anti-human CD19, clone HIB19; KIRAVIA Blue 520 anti-human CD3, clone UCHT1; PE anti-human CD56 (NCAM) recombinant, clone QA17A16; Zombie Aqua Fixable Viability Dye.
